# Framework Uranyl Silicates: Crystal Chemistry and a New Route for the Synthesis

**DOI:** 10.3390/ma16114153

**Published:** 2023-06-02

**Authors:** Evgeny V. Nazarchuk, Oleg I. Siidra, Dmitri O. Charkin, Yana G. Tagirova

**Affiliations:** 1 Department of Crystallography, Saint-Petersburg State University, University emb. 7/9, 199034 St. Petersburg, Russia; o.siidra@spbu.ru (O.I.S.); yanki.tagirova@yandex.ru (Y.G.T.); 2 Kola Science Center, Russian Academy of Sciences, 184200 Apatity, Russia; 3 Department of Chemistry, Moscow State University, Vorobievy Gory 1, bd. 3, 119991 Moscow, Russia

**Keywords:** uranyl compounds, silicates, microporous structures, inorganic synthesis

## Abstract

To date, uranyl silicates are mostly represented by minerals in nature. However, their synthetic counterparts can be used as ion exchange materials. A new approach for the synthesis of framework uranyl silicates is reported. The new compounds Rb_2_[(UO_2_)_2_(Si_8_O_19_)](H_2_O)_2.5_ (**1**), (K,Rb)_2_[(UO_2_)(Si_10_O_22_)] (**2**), [Rb_3_Cl][(UO_2_)(Si_4_O_10_)] (**3**) and [Cs_3_Cl][(UO_2_)(Si_4_O_10_)] (**4**) were prepared at harsh conditions in “activated” silica tubes at 900 °C. The activation of silica was performed using 40% hydrofluoric acid and lead oxide. Crystal structures of new uranyl silicates were solved by direct methods and refined: **1** is orthorhombic, *Cmce*, *a* = 14.5795(2) Å, *b* = 14.2083(2) Å, *c* = 23.1412(4) Å, *V* = 4793.70(13) Å^3^, *R*1 = 0.023; **2** is monoclinic, *C*2/*m*, *a* = 23.0027(8) Å, *b* = 8.0983(3) Å, *c* = 11.9736(4) Å, *β* = 90.372(3) °, *V* = 2230.43(14) Å^3^, *R*1 = 0.034; **3** is orthorhombic, *Imma*, *a* = 15.2712(12) Å, *b* = 7.9647(8) Å, *c* = 12.4607(9) Å, *V* = 1515.6(2) Å^3^, *R*1 = 0.035, **4** is orthorhombic, *Imma*, *a* = 15.4148(8) Å, *b* = 7.9229(4) Å, *c* = 13.0214(7) Å, *V* = 1590.30(14) Å^3^, *R*1 = 0.020. Their framework crystal structures contain channels up to 11.62 × 10.54 Å filled by various alkali metals.

## 1. Introduction

The rising interest in studies of hexavalent uranium silicates is underpinned by a variety of reasons, including the mineralogy of the oxidation areas of uranium deposits [1] and the technogenesis of spent nuclear fuel (SNF) [2]. By now, this family hosts 21 mineral species [3], as well as ca. 40 synthetic compounds containing uranium and silicon. Natural uranyl silicates are formed at the earlier formation stages of the oxidation areas of uranium deposits. Due to their ability to exchange cations, as exemplified by boltwoodite, (K,Na)[(UO_2_)(SiO_3_OH)](H_2_O)_1.5_ [4] and cuprosklodowskite, Cu[(UO_2_)_2_(SiO_3_OH)_2_](H_2_O)_6_ [5], uranyl silicates are expected to take an active and essential part in the migration, accumulation, and deposition processes.

Model experiments on oxidation of UO_2_ [6] and the hydration of uranium-doped borosilicate glasses [7] have demonstrated the formation of uranyl silicates during SNF oxidation. Structural peculiarities of a KNa_3_[(UO_2_)_2_(Si_4_O_10_)_2_](H_2_O)_4_ compound obtained during glass hydration suggest it to be a potential absorber of radionuclides, such as Np^V^ or Am^III^. The promising properties of uranyl silicates, both natural and synthetic, arise from their highly porous crystal structures wherein the uranyl cation generally coordinates four to five ligands in the equatorial plane (most commonly oxygen atoms from oxyanions, hydroxyl groups, water molecules, and halide anions) with formation of tetra- or pentagonal bipyramids. These polyhedra generally do not share edges or vertices, except for the representatives of the uranophane group [8] and layers in the haiweeite structure [9].

**Figure 1:**
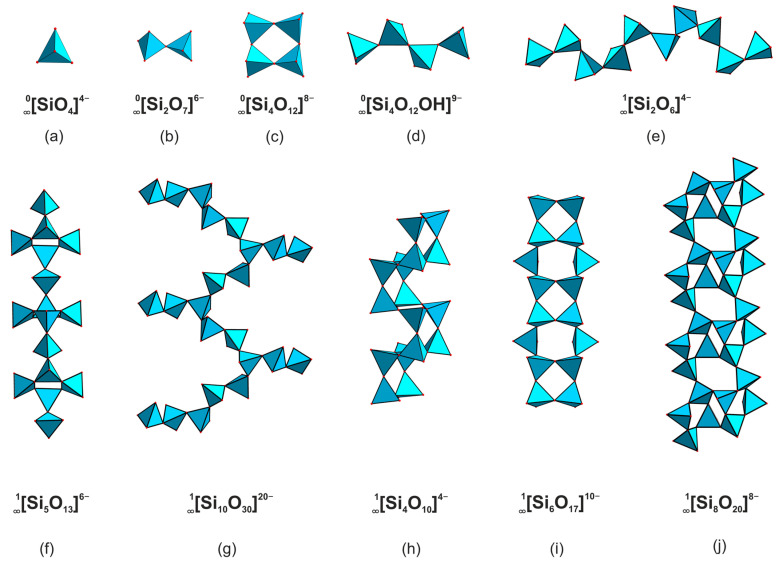
Isolated and chain complexes in the structures of uranyl silicates. The $\begin{array}{c} 0\\ \infty \end{array}$[SiO_4_]^4−^ (**a**) in the structure of, for example, α-uranophane [20], $\begin{array}{c} 0\\ \infty \end{array}$ [Si_2_O_7_]^6−^ (**b**) in Na_6_[(UO_2_)_3_(Si_2_O_7_)_2_] [21], $\begin{array}{c} 0\\ \infty \end{array}$ [Si_4_O_12_]^8−^ (**c**) in [Cs_9_Cs_6_Cl][(UO_2_)_7_(Si_6_O_17_)_2_(Si_4_O_12_)] [14] and $\begin{array}{c} 0\\ \infty \end{array}$ [Si_4_O_12_OH]^9−^ (**d**) in K_5_[(UO_2_)_2_(Si_4_O_12_(OH))] [12]. Linear $\begin{array}{c} 1\\ \infty \end{array}$ [Si_2_O_6_]^4−^ (**e**) in K_2_[(UO_2_)Si_2_O_6_] [19] or $\begin{array}{c} 1\\ \infty \end{array}$ [Si_5_O_13_]^6−^ (**f**) in haiweeite [9], branched $\begin{array}{c} 1\\ \infty \end{array}$ [Si_10_O_30_]^20−^ (**g**) in K_14_[(UO_2_)_3_Si_10_O_30_] [19], tubular, and multi-layer chains like $\begin{array}{c} 1\\ \infty \end{array}$ [Si_4_O_10_]^4−^ (**h**) in [Cs_3_F][(UO_2_)(Si_4_O_10_)] [21] and $\begin{array}{c} 1\\ \infty \end{array}$ [Si_6_O_17_]^10−^ (**i**) in [Cs_2_Cs_5_F][(UO_2_)_2_(Si_6_O_17_)] [14], as well as $\begin{array}{c} 1\\ \infty \end{array}$ [Si_8_O_20_]^8−^ (**j**) in Rb_4_[(UO_2_)(Si_8_O_20_)] [18].

**Figure 2:**
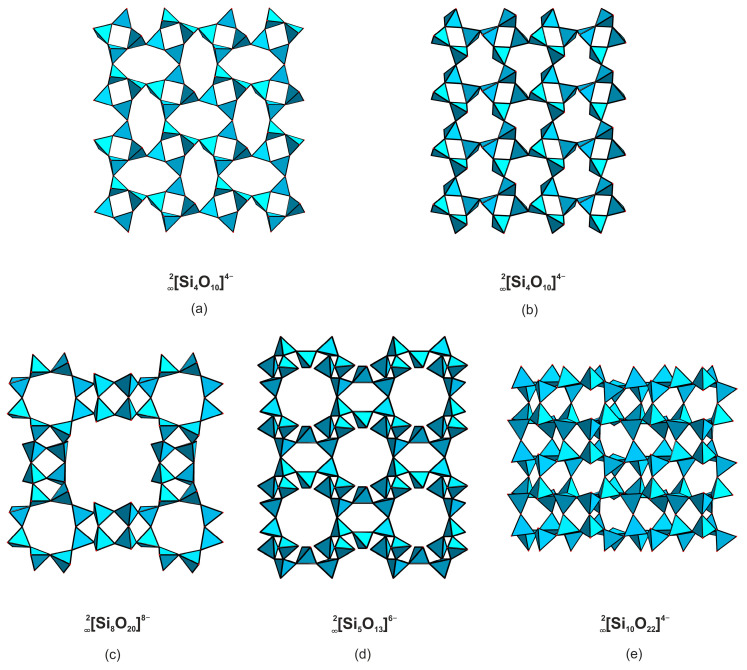
Layered complexes in the structures of uranyl silicates. The $\begin{array}{c} 2\\ \infty \end{array}$ [Si_4_O_10_]^4−^ (**a**) in the structure of KNa_3_[(UO_2_)_2_(Si_4_O_10_)_2_](H_2_O)_4_ [7], $\begin{array}{c} 2\\ \infty \end{array}$[Si_4_O_10_]^4−^ (**b**) in β-K_2_[(UO_2_)(Si_4_O_10_)] [21], $\begin{array}{c} 2\\ \infty \end{array}$[Si_8_O_20_]^8−^ (**c**) in K_4_[(UO_2_)_2_(Si_8_O_20_)](H_2_O)_4_ [21], $\begin{array}{c} 2\\ \infty \end{array}$[Si_5_O_13_]^6−^ (**d**) in weeksite [22], and $\begin{array}{c} 2\\ \infty \end{array}$[Si_10_O_22_]^4−^ (**e**) in Cs_2_[(UO_2_)Si_10_O_22_] [23].

In the structures of most natural uranyl silicates, excluding weeksite group, calcioursilite Ca_4_[(UO_2_)_4_(Si_2_O_5_)_5_(OH)_6_](H_2_O)_15_ [10], and magnioursilite Mg_4_[(UO_2_)_4_(Si_2_O_5_)_5_(OH)_6_](H_2_O)_20_ [11], the silicate sublattice is represented by just the monomeric orthosilicate tetrahedra, $\begin{array}{c} 0\\ \infty \end{array}$[SiO_4_]^4−^. In contrast, the structures of synthetic compounds are more diverse and host four 0*D*, six 1*D*, and five 2*D* architectures (Figure 1, Figure 2 and Table S1). The former is represented by the $\begin{array}{c} 0\\ \infty \end{array}$[SiO_4_]^4−^ (Figure 1a), $\begin{array}{c} 0\\ \infty \end{array}$[Si_2_O_7_]^6−^ (Figure 1b), $\begin{array}{c} 0\\ \infty \end{array}$[Si_4_O_12_]^8−^ (Figure 1c), and $\begin{array}{c} 0\\ \infty \end{array}$[Si_4_O_12_OH]^9−^ (Figure 1d) species where two or more can be present within the same structure. The structure of K_5_[(UO_2_)_2_(Si_4_O_12_(OH))] [12] contains relatively rare protonated silicate anions which align in chains due to hydrogen bonding.

Condensation of the monomeric tetrahedra via vertex sharing leads to formation of linear $\begin{array}{c} 1\\ \infty \end{array}$[Si_2_O_6_]^4−^ (Figure 1e) or $\begin{array}{c} 1\\ \infty \end{array}$[Si_5_O_13_]^6−^ (Figure 1f), branched $\begin{array}{c} 1\\ \infty \end{array}$[Si_10_O_30_]^20−^ (Figure 1g), tubular, and multi-layer chains, such as $\begin{array}{c} 1\\ \infty \end{array}$[Si_4_O_10_]^4−^ (Figure 1h) and $\begin{array}{c} 1\\ \infty \end{array}$[Si_6_O_17_]^10−^ (Figure 1i), as well as $\begin{array}{c} 1\\ \infty \end{array}$[Si_8_O_20_]^8−^ ribbons (Figure 1j) [13,14,15,16,17,18]. As in the previous case, a single structure can host polymeric anions with different compositions and architectures [19].

Five layered silicate anions have been reported in the structures of uranyl compounds: two isomers of $\begin{array}{c} 2\\ \infty \end{array}$[Si_4_O_10_]^4−^ (Figure 2a,b), $\begin{array}{c} 2\\ \infty \end{array}$[Si_8_O_20_]^8−^ (Figure 2c), $\begin{array}{c} 2\\ \infty \end{array}$[Si_5_O_13_]^6−^ (Figure 2d), and $\begin{array}{c} 2\\ \infty \end{array}$[Si_10_O_22_]^4−^ (Figure 2e). The topology of the two latter will be discussed below in detail. Two isomers of $\begin{array}{c} 2\\ \infty \end{array}$[Si_4_O_10_]^4−^, present in the structures of Na_2_[(UO_2_)(Si_4_O_10_)](H_2_O)_2.1_ [15] and anhydrous K_2_[(UO_2_)(Si_4_O_10_)] [21], are built of vertex-sharing [Si_4_O_12_]^8−^ groups; they differ in bond angles between the SiO_4_ tetrahedra and orientation matrices of the terminal vertices. A silicate layer containing two types of rings can be illustrated by the $\begin{array}{c} 2\\ \infty \end{array}$[Si_8_O_20_]^8−^ architecture (Figure 2c) in the structure of K_4_[(UO_2_)_2_(Si_8_O_20_)](H_2_O)_4_ [21], which is comprised of condensed four- and eight-membered cycles.

Among uranyl silicates, the UO*_n_* and SiO_4_ polyhedra commonly condense into microporous frameworks [24]. Though such architectures are known also among uranyl molybdates [25,26,27,28,29], selenates [30], chromates [31], vanadates [32,33], phosphates [34,35], sulfates, [36,37,38] and phosphonates [39], the silicate frameworks exhibit more complex and uncommon topologies. Comparable architectures have by now been reported only among uranyl germanates [40,41].

Several approaches to synthesis of framework uranyl silicates are known including “soft” [18] and “hard” [17,42] hydrothermal treatment, as well as salt flux synthesis [40]. Both standard Teflon-lined autoclaves [42], and sealed gold tubes [18,43] have been used for the “soft” and “hard” hydrothermal processes, respectively. In the high-temperature syntheses, use of fluxing agents, mostly molten alkali metal halides, permits obtaining the so-called salt-inclusion structures, which can be classified as microporous zeolite-like frameworks [14,44].

In our experiments, we employed new techniques which permitted preparation of single crystals of four new uranyl silicates: Rb_2_[(UO_2_)_2_(Si_8_O_19_)](H_2_O)_2.5_ (**1**), (K,Rb)_2_[(UO_2_)(Si_10_O_22_)] (**2**), [Rb_3_Cl][(UO_2_)(Si_4_O_10_)] (**3**), and [Cs_3_Cl][(UO_2_)(Si_4_O_10_)] (**4**), which are described below.

## 2. Experimental

*Caution*! *Although the uranium precursors used contain depleted uranium*, *standard safety measures for handling radioactive substances must be followed*.

### 2.1. Synthesis

**Figure 3:**
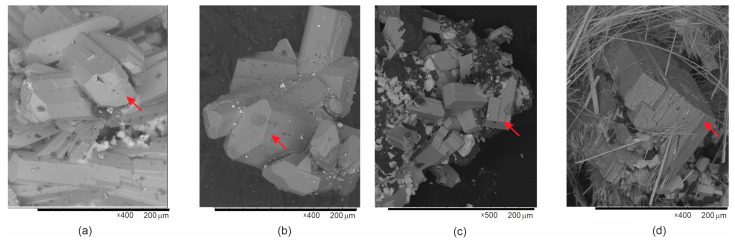
SEM images of Rb_2_[(UO_2_)_2_(Si_8_O_19_)](H_2_O)_2.5_ (**a**), (K,Rb)_2_[(UO_2_)(Si_10_O_22_)] (**b**), [Rb_3_Cl][(UO_2_)(Si_4_O_10_)] (**c**) and [Cs_3_Cl][(UO_2_)(Si_4_O_10_)] (**d**) crystals. Red arrows point to crystals 1–4.

**Rb_2_**[(**UO_2_**)**_2_**(**Si_8_O_19_**)](**H_2_O**)**_2_**_.**5**_. Yellow platelets of **1** (Figure 3a) were obtained in a high-temperature synthesis. The starting compounds were 135 mg U_3_O_8_ (Vecton, Russia, 99.7%), 24 mg of RbCl (Vecton, 99.7%), 67 mg of PbO (Vecton, 99.7%). As some previous experiments have indicated that the reaction between uranium and silicon oxides requires harsh conditions, the reagents were additionally activated. The mixture was transferred into a silica tube (which served also as the source of silicon), then 30 μL of 40% hydrofluoric acid was injected. After one minute, the tube was attached to a vacuum line, evacuated, and sealed. The tube was heated to 900 °C at a rate of 100 °C/h, annealed for 50 h, and cooled to room temperature at the rate of 10 °C/h. Reaction with the tube walls produced the target crystals. Tiny drops of solidified Pb metal were also found in the sample indicating oxidation of U_3_O_8_ by the lead oxide during reaction.

(**K**,**Rb**)**_2_**[(**UO_2_**)(**Si_10_O_22_**)]. The crystals of **2** (Figure 3b) were obtained via the same protocol but starting from 37 mg of RbCl (Vecton, 99.7%), 37 mg of KCl (Vecton, 99.7%), 252 mg of U_3_O_8_ (Vecton, 99.7%), and 18 mg of SiO_2_ (Vecton, 99.7%).

**Figure 4:**
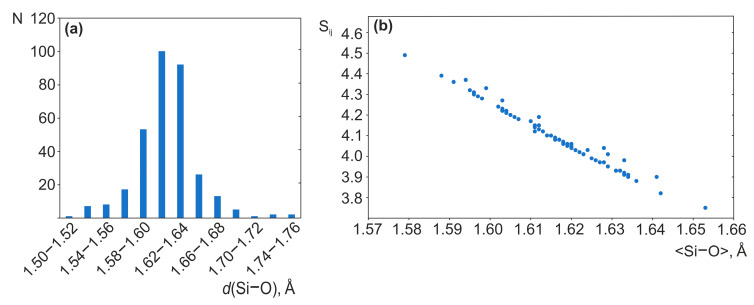
The distribution of occurrence of the respective Si-O bond lengths in the structures of uranyl silicates (**a**), and the dependence of BVS for silicon atoms on the mean distance to oxygens (**b**).

[**Rb_3_Cl**][(**UO_2_**)(**Si_4_O_10_**)] and [**Cs_3_Cl**][(**UO_2_**)(**Si_4_O_10_**)]. Yellow prismatic crystals of **3** (Figure 3c) and **4** (Figure 3e) were also produced in high-temperature experiments. Mixtures of 48 mg of RbCl (CsCl) (Vecton, 99.7%), 54 mg U_3_O**_8_** (Vecton, 99.7%), and 90 mg PbO (Vecton, 99.7) were pre-dried at 80 °C and annealed in “activated” silica tubes and processed as described above.

### 2.2. Crystal Structure Determination

Single crystals of **1**–**4** selected for X-ray diffraction analysis were attached onto glass fibers and mounted on a Rigaku XtaLAB Synergy-S diffractometer (Tokyo, Japan) equipped with a PhotonJet-S detector (Tokyo, Japan) operating with MoKα radiation at 50 kV and 1 mA. More than a hemisphere of data was collected in each case with a frame width of 0.5° in ω, and counting time of 10 s. The data were integrated and corrected for absorption applying a multiscan type model using the Rigaku Oxford Diffraction programs CrysAlis Pro (Rigaku OD, 2015) (Tokyo, Japan). The experiments were performed with cooling to 150 K. The unit cell parameters were calculated by the least-squares method. The structures were solved u direct methods using WinGX (Glasgow, UK) [45] and Olex2 (Regensburg, Germany) [46] software. The main parameters of the experiment and refinement are collected in Table 1. The final solutions include the coordinates and anisotropic thermal parameters of atoms. Selected interatomic distances are collected in Tables S2–S5.

### 2.3. Characterization

Powder X-ray diffraction patterns (PXRD) were recorded on a Rigaku R-AXIS RAPID diffractometer (Tokyo, Japan) utilizing CoKα radiation operating at 50 kV and 10 mA. Simulated PXRD patterns were calculated from single-crystal data using the Diamond program (Bonn, Germany) (Figures S1 and S2). The infrared (IR) spectra were measured on a Bruker vertex 70 spectrometer (Ettlingen, Germany) in the range of 4000–400 cm^−1^ from samples pressed into KBr pellets (Figure S3). Microprobe analysis was performed on a Hitachi S-3400N SEM (Tokyo, Japan) with analytical devices: with analytical chamber: EBSD–AzTec HKL Channel 5 Advanced, quantitative EDX–AzTec Energy 350, quantitative WDS–INCA 500 and using the standards listed in Tables S6–S9.

## 3. Results

**Rb_2_[(UO_2_)_2_(Si_8_O_19_)](H_2_O)_2.5_** (**1**)

In the crystal structure of **1**, the uranium atom forms a uranyl cation (<U-O_ap_> = 1.806 Å) which is coordinated, in the equatorial plane, by five oxygen atoms (<U-Oeq> = 2.370 Å). Four symmetrically independent silicon atoms are tetrahedrally coordinated with <Si-O> = 1.603–1.617 Å. Two symmetry independent rubidium cations are coordinated by oxygen atoms, including those from four water molecules (<Rb-O> = 2.908, 2.938 Å). To analyze the coordination environment and estimate the valence states, bond valence calculations were performed according to [47]. Full details of the bond valence model can be found in [48]. The bond valence sums are 5.91, 4.16, 4.07, 4.19, 4.22 for U1, and Si1–Si4, respectively (Table S10). The slight overbonding for the silicon atoms is rather commonly observed among the structures of uranyl silicates (Table S11). For instance, the BVS for Si1 and Si2 in β-K_2_[(UO_2_)(Si_4_O_10_)] are 4.39 and 4.31, respectively, while for K_4_[(UO_2_)_2_(Si_8_O_20_)](H_2_O)_4_ [21] and Cs_2_[(UO_2_)(Si_10_O_22_)] [23], the BVS for the silicon atoms line is in a broad range of 3.90–4.53. According to the formula given in [49], these values strongly and almost linearly depend on the bond distances in the SiO_4_ tetrahedra. Among uranyl silicates, the distribution of these values is close to normal with the maximum at 1.60–1.62 Å (Figure 4a). The BVS range of 4.0 ± 0.1 is attained at *d*(SiO) = 1.615–1.635 Å (Figure 4b), while the ideal value of 4.0, at *d*(Si-O) = 1.622 Å. The range of 1.60–1.62 Å corresponds to BVS range of 4.05–4.25 *v.u.* while the quite abundant values of 1.62–1.64 Å yield BVS of 4.05–3.85 *v.u.* In certain cases, mean bond distances of 1.56–1.60 Å are reported, so that BVS exceeds 4.25, as well as 1.64–1.66 Å, when the BVS drops below 3.85. The deviations of bond valence sum for silicon from the ideal value of 4.0 are due to the distortions of ideal regular SiO_4_ tetrahedra during the formation of polysilicate complexes when certain (terminal) bonds shorten down to 1.50 Å.

**Figure 5:**
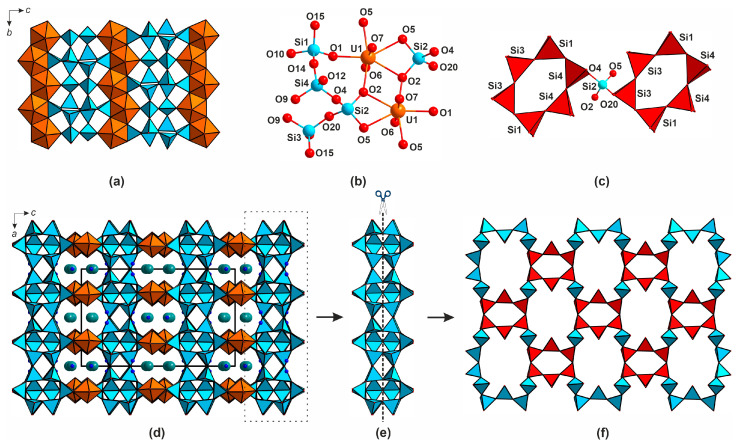
The crystal structure of Rb_2_[(UO_2_)_2_(Si_8_O_19_)](H_2_O)_4_ (**1**). Linkage of the uranium and silicon polyhedra in traditional (**a**) and ball-and-stick representation (**b**). Linkage of [Si_6_O_18_]^12−^ rings via the Si2O_4_ tetrahedron (**c**). Projection of the structure **1** onto *ac* (**d**). The double $\begin{array}{c} 2\\ \infty \end{array}$[Si_8_O_19_]^6−^ layers and their dissection (**e**) and the structure of the “single” layers (**f**). Uranium polyhedra are shown in brown, and silicon in blue. The 6-membered rings [Si_6_O_18_]^12−^ are highlighted in red. Rubidium atoms are shown in dark green, and water molecules are dark blue.

In the structure of **1**, the UO_7_ polyhedra condense to form $\begin{array}{c} 1\\ \infty \end{array}$[UO_5_]^4−^ chains (Figure 5a) which share vertices and edges with the SiO_4_ tetrahedra (Figure 5b). The latter associate into 6-membered rings (Figure 5c) and further into $\begin{array}{c} 2\\ \infty \end{array}$[Si_8_O_19_]^6−^ layers, which are stitched by the uranium polyhedra into a microporous framework (Figure 5d). The channels (9.15 × 7.31 Å) host the rubidium cations and water molecules.

Dissection of the $\begin{array}{c} 2\\ \infty \end{array}$[Si_8_O_19_]^6−^ layers in a way shown in Figure 5e yields a $\begin{array}{c} 2\\ \infty \end{array}$[Si_4_O_11_]^6**−**^ layer comprised of 6-membered [Si_6_O_18_]^12−^ rings stitched by Si2O_4_ tetrahedra (Figure 5f). The $\begin{array}{c} 2\\ \infty \end{array}$[Si_8_O_19_]^6−^ layers (Figure 5e) can be described as being formed via the coalescence of two $\begin{array}{c} 2\\ \infty \end{array}$[Si_4_O_11_]^6−^ layers. In other words, the layers in the structures of **1** can be traced to the [Si_4_O_11_]^6−^ “building blocks”. The silicate layers in **1** are comprised of 6-membered [Si_6_O_18_]^12**−**^ rings, which are linked by single SiO_4_ tetrahedra (Figure 5c).

**Figure 6:**
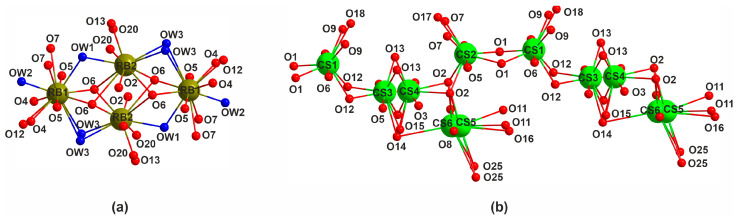
Coordination of alkali metal cations and the ways of their linkage in the structures of **1** (**a**) and Cs_2_[(UO_2_)_2_(Si_8_O_19_)] (**b**). Rubidium atoms are shown in dark green, water molecules are dark blue, and cesium atoms are shown in green, oxygen—red.

A compound Cs_2_[(UO_2_)_2_(Si_8_O_19_)] with a framework similar to **1** has been reported in [24]. Both **1** and Cs_2_[(UO_2_)_2_(Si_8_O_19_)] exhibit very similar cell parameters (*a* = 14.5795(2) vs. 14.1955(2) Å, *b* = 14.2083(2) vs. 14.6274(2) Å, *c* = 23.1412(4) vs. 23.0639(4) Å, *V* = 4793.70(13) vs. 4789.06(13)Å^3^), the differences being mostly due to the replacement of a larger Cs^+^ by a smaller Rb^+^ and the presence of water molecules. The framework topology in both compounds is nearly identical; the most pronounced differences concern the positions and coordination of the alkali metal cations (Figure 6).

In **1**, the rubidium cations occupy two ordered sites. Their polyhedra share edges to form tetrameric complexes (Figure 6a). In the structure of Cs_2_[(UO_2_)_2_(Si_8_O_19_)], four out of the six cesium sites are disordered (Figure 6b). The Cs3 and Cs4 have 70/30%, while Cs5 and Cs6, 75/25% occupancies. The cesium polyhedra also share edges, but to form twisted chains.

**Figure 7:**
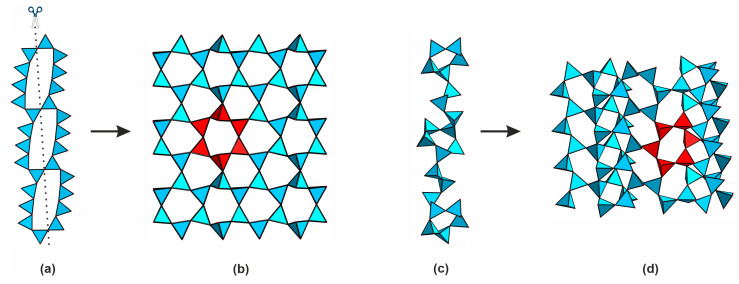
Double $\begin{array}{c} 2\\ \infty \end{array}$[Si_8_O_19_]^6−^ layers in the structures of Cs_2_Cu_2_(Si_8_O_19_) and Rb_2_Cu_2_(Si_8_O_19_) (**a**) and the $\begin{array}{c} 2\\ \infty \end{array}$ [Si_4_O_10_]^4−^ layers obtained upon their dissection (**b**). The alternative $\begin{array}{c} 2\\ \infty \end{array}$ [Si_8_O_19_]^6−^ arrangement in the structure of Na_6_(Si_8_O_19_) (**c**,**d**). Silicon polyhedra are shown in blue. The dashed line reflects the dissection direction; the [Si_6_O_18_]^12−^ units are highlighted in red.

Two more topologies of the $\begin{array}{c} 2\\ \infty \end{array}$[Si_8_O_19_]^6−^ chains are known, both comprised of the [Si_6_O_18_]^12−^ hexameric “building blocks”. The former is found in the structures of Cs_2_Cu_2_(Si_8_O_19_) [50] and Rb_2_Cu_2_(Si_8_O_19_) [51], while the second, is found in the structure of Na_6_(Si_8_O_19_) [52]. In the structures of the copper silicates, the silicate tetrahedra share vertices to form the $\begin{array}{c} 2\\ \infty \end{array}$[Si_8_O_19_]^6−^ layers shown in Figure 7a. Dissecting them (Figure 7a) and rotating one-half results in the formation of $\begin{array}{c} 2\\ \infty \end{array}$[Si_4_O_10_]^4−^ well known in micas and other layered clay minerals (Figure 7b). The other layer type (Figure 7c) has a more complex “step-lattice” arrangement (Figure 7d). Yet, the [Si_6_O_18_]^12−^ building blocks are clearly visible in both architectures.

**(K,Rb)_2_[(UO_2_)(Si_10_O_22_)]** (**2**).

**Figure 8:**
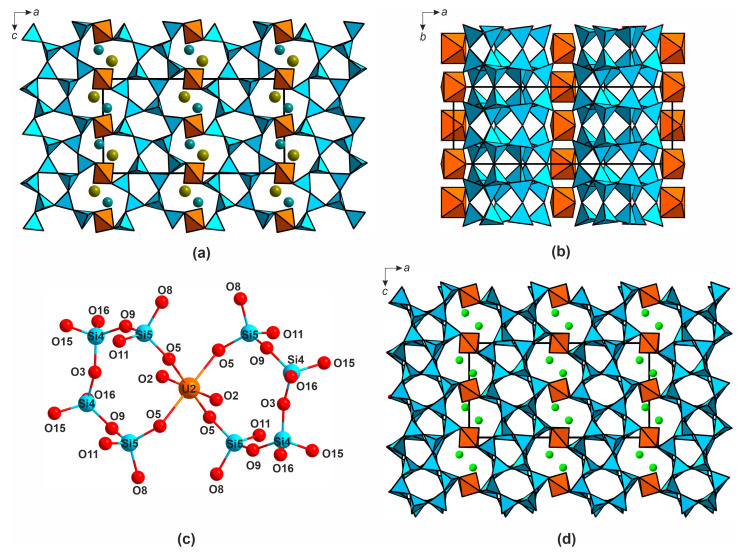
Projections of the crystal structure of (K,Rb)_2_[(UO_2_)(Si_10_O_22_)] (**2**) onto *ac* (**a**) and *ab* (**b**) planes. The linkage of UO_6_ and SiO_4_ polyhedra (**c**). *ac*-Projection of the structure of Cs_2_[(UO_2_)(Si_10_O_22_)] (**d**). The colors are the same as in Figure 5.

In the crystal structure of **2**, there are two symmetry independent uranium sites corresponding to uranyl cations (<U-O_ap_> = 1.798, 1.794 Å). These are coordinated, in the equatorial planes, by four oxygen atoms to form tetragonal bipyramids (<U-O_eq_> = 2.275, 2.269 Å). Five silicon sites are tetrahedrally coordinated (<Si-O> = 1.585–1.611 Å). The alkali metal cations positions are ten- and nine-coordinated. The bond valence sums are 5.84, 5.90, 4.20, 4.16, 4.43, 4.42, 4.14 for U1, U2, and Si1-Si5, respectively (Table S12). In the structure of **2** (Figure 8a,b), the SiO_4_ tetrahedra condense into $\begin{array}{c} 2\\ \infty \end{array}$[Si_10_O_22_]^4−^ double layers which are stitched by the UO_6_ bipyramids (Figure 8c) into a porous framework (Figure 8d).

In contrast to **1**, in **2** the uranium polyhedra do not condense but share their equatorial vertices only with the silicate tetrahedra SiO_4_. The channels, of 7.69 × 4.38 Å width, contain alkali metal cations.

**Figure 9:**
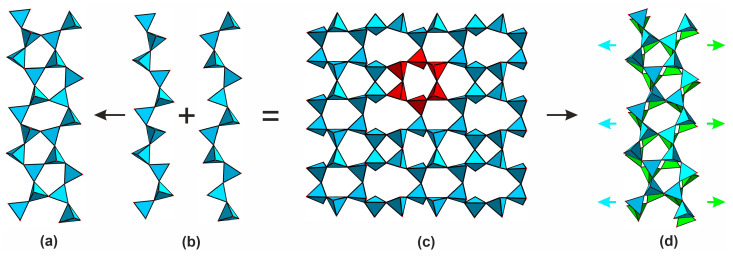
The double $\begin{array}{c} 2\\ \infty \end{array}$[Si_10_O_22_]^4−^ layer in the structure of **2** (**a**) and the way of its dissection (**b**). Red [Si_6_O_18_]^12−^ rings in the structure of [Si_10_O_22_]^4−^ in **2** (**c**). Variation of their geometry when passing from 2 to Cs_2_[(UO_2_)(Si_10_O_22_)] (**d**), the “shifted” part is highlighted in green. Silicon polyhedra are shown in blue.

The double $\begin{array}{c} 2\\ \infty \end{array}$[Si_10_O_22_]^4−^ layers (Figure 9a) can also be dissected, as shown in Figure 9b. In this case, the dissection produces layers which also contain the [Si_6_O_18_]^12−^ building blocks (Figure 9c). The compound Cs_2_[(UO_2_)(Si_10_O_22_)] [23] is characterized by very similar unit cell parameters (after the corresponding interchange) and positions of heavy atoms, compared to **2** (*a* = 23.0027(8) vs. 23.3796(8) Å (interchanged), *b* = 8.0983(3) vs. 8.0518(3) Å, *c* = 11.9736(4) vs. 12.2506(4) Å (interchanged), *β* = 90.372(3) vs. 90.011(2) °, *V* = 2230.43(14) vs. 2306.15(11) Å^3^, for **2** and the cesium compound, respectively). The differences are evidently due to smaller size of Rb^+^ and K^+^ compared to Cs^+^. It is probably also the reason for the differences in symmetry (*C*2/*m* for **2** and *P*2_1_/*c* for Cs_2_[(UO_2_)(Si_10_O_22_)]. We attempted to solve the structure of **2** in the *P*2_1_/*c* by analogy but failed (*R*1 ≥ 20%, unstable refinement of light atoms). The arrangements of the silicon, uranium, and alkali metal cations in *C*2/*m* and *P*2_1_/*c* are nearly the same, and the difference between the two structures is in the geometry of the double $\begin{array}{c} 2\\ \infty \end{array}$[Si_10_O_22_]^4−^ layers (Figure 9a,d).

In both structures, double $\begin{array}{c} 2\\ \infty \end{array}$[Si_10_O_22_]^4−^ layer can be dissected into ribbons; in **2**, they are aligned one against other as dictated by a mirror plane, so one ribbon is completely covered by another on the corresponding projection (Figure 9a). In the meantime, in the structure of Cs_2_[(UO_2_)(Si_10_O_22_)] the ribbons are shifted due to the rotations of SiO_4_ tetrahedra (Figure 9d), resulting in a decrease of the overall symmetry.

**[Rb_3_Cl][(UO_2_)(Si_4_O_10_)]** (**3**) and **[Cs_3_Cl][(UO_2_)(Si_4_O_10_)]** (**4**)

**Figure 10:**
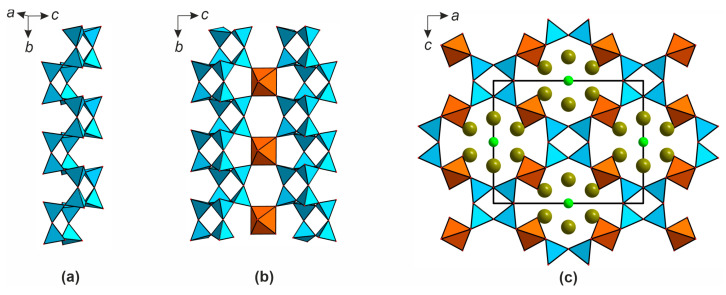
The crystal structures of [*A*_3_Cl][(UO_2_)(Si_4_O_10_)], (*A* = Rb, Cs) (**3**, **4**). The $\begin{array}{c} 1\\ \infty \end{array}$[Si_4_O_10_]^4−^ chains (**a**), their linkage by the UO_6_ bipyramids (**b**), and the corresponding *ac* projection (**c**). The colors are the same as in Figure 5.

The crystal structures of **3** and **4** contain a single uranium site forming a uranyl cation (<U-O_ap_> = 1.801 and 1.805 Å for **3** and **4**, respectively). As in the previous case, these species coordinate four oxygen atoms in equatorial planes (<U-O_eq_> = 2.252 and 2.261 Å for **3** and **4**). A unique silicon site centers a tetrahedron (<Si-O> = 1.605 and 1.609 Å for **3** and **4**). The bond valence sums are 5.94, 4.18, and 5.94, 4.20 for U1, Si1 in the structure of **3** and **4**, respectively (Tables S13 and S14). The SiO_4_ tetrahedra share vertices to form $\begin{array}{c} 1\\ \infty \end{array}$[Si_4_O_10_]^4−^ chains aligned along *b* (Figure 10a) and linked into framework by the uranium polyhedra (Figure 10b,c).

The length of the equatorial edge of the UO_6_ bipyramid nearly coincides with the distance between the oxygen atoms in the silicate chain, which permits these bipyramids to reside at the bending points of the zigzag chains (Figure 10b). The channels of framework with the interior size of 11.62 × 10.54 Å are aligned in the *ac* plane and contain chloride anions and alkali metal cations (Rb^+^ in **3** and Cs^+^ in **4**). Such salt-inclusion structures are rather common among uranyl silicates [44,53], with halide anions either being coordinated to the uranyl cations [54] or filling the channels. Booth **3** and **4** are isostructural to the fluoride silicate [Cs_3_F][(UO_2_)(Si_4_O_10_)] [21]. It is noteworthy that despite essential difference in the ionic radii of F^−^ and Cl^−^ and more reactive bonding of the former to uranyl cations, both halide anions contribute to isostructural compounds.

## 4. Discussion

**Figure 11:**
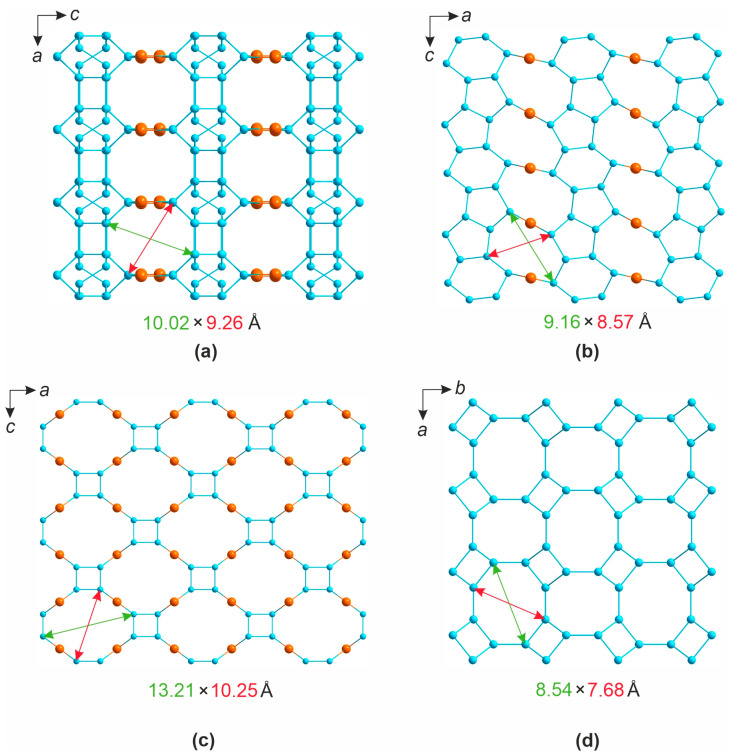
The graphs for the frameworks in Rb_2_[(UO_2_)_2_(Si_8_O_19_)](H_2_O)_2.5_ (**a**), (K,Rb)_2_[(UO_2_)(Si_10_O_22_)] (**b**), [Rb_3_Cl][(UO_2_)(Si_4_O_10_)] and Cs_3_Cl(UO_2_)(Si_4_O_10_) (**c**) and merlinoite K_6_Ca_2_[Al_10_Si_22_O_64_]·20H_2_O (**d**). The channel metrics are indicated below for each case. Uranium atoms are shown in brown, silicon in blue.

Consider now the graphs of the frameworks in the structures of **1**–**4**, which are produced by omitting the alkali metal cations, halide anions, and oxygen atoms, including those from water molecules, and joining the nodes formed by uranium and silicon atoms whose polyhedra share vertices or edges. The results reflect the connectivity modes (Figure 11). The channel dimensions, measured as the internodal distances, are relatively close. The graphs for **1** (Figure 11a) contain six- and eight-membered rings. The graph topology for **1** are rather close to those reported for the structure of zeolite merlinoite, K_6_Ca_2_[Al_10_Si_22_O_64_]·20H_2_O [55,56] (Figure 11d).

Considering these related structures, we conclude that this framework tolerates both the replacement of K^+^ by Rb^+^ and the variation of the water content in the channels. This suggests that some exchange properties may be expected. These features are even more pronounced for the frameworks in **1**–**4**. In **1**, the channels contain Rb^+^ cations and water molecules, while in Cs_2_[(UO_2_)_2_(Si_8_O_19_)] [24], only Cs^+^ cations. The framework in **2** is also rather “elastic”: its channels can be filled by Cs^+^, K^+^/Rb^+^ and water molecules. The framework in **3** and **4** remains stable upon replacement of Cs^+^ by Rb^+^ and F^−^ by Cl^−^. Considering that the size difference between F^−^ and Cl^−^ is essentially larger than that between Cl^−^ and Br^−^, existence of the corresponding bromide analogs, at least with small- and medium-size alkali metal cations and maybe even Ag^+^ and Tl^+^, does not seem unlikely; at least some of these species can likely be prepared via cation/anion exchange using low-temperature eutectic halide melts, which is of certain interest considering immobilization of ^137^Cs^+^ or ^36^Cl^−^.

The compounds **1**–**4** were prepared at harsh conditions (900 °C). Despite the gross differences in preparation conditions, they share some common structural features: for instance, their silicate architectures contain [Si_6_O_18_]^12−^ building blocks. It needs to be noted that while the silicon source in the hydrothermal syntheses is very likely the very reactive form of dissolved silica, in high-temperature syntheses it is the glassy form of SiO_2_ which is also rather reactive compared to its crystalline forms, particularly quartz. Yet, the presence of some activators, such as PbO or HF or alkali fluorides, which react with silica at much lower temperatures, compared to uranium oxides, is also important. The activation is either due to the attack of the initially smooth and less reactive silica surface, or via the formation of volatile and reactive species, such as SiF_4_ or UO_2_F_2_; note, however, that the exact sequence of reactions is obscure (neither activator is incorporated into the uranyl silicates reported here). The role of PbO is also in oxidation of U_3_O_8_ into U^VI^ compounds. This suggests that certain silicate species are either most easily formed during synthesis or exhibit exceptional stability to occur under totally dissimilar synthesis conditions. The possible templating role of alkali metal and uranyl cations in formation of complex silicate architectures under hydrous and (nearly) anhydrous conditions is also an open and appealing question. The structural similarities and dissimilarities between silicates, germanates, and some more distant relatives, such as alumo- or gallophosphates are also of essential interest. Investigations aimed at finding at least primary and partial answers to these questions are currently underway.

## 5. Conclusions

Four novel uranyl–alkali metal silicates have been synthesized via high-temperature synthesis in evacuated “activated” silica tubes using PbO as an oxidizer and fluxing agent. Their crystal structures can be described as frameworks containing channels with effective radii of up to 11.62 × 10.54 Å; these are filled by alkali metal cations and water molecules. These frameworks are relatively stable against substitution in the cation sublattice and variation of water content; this indicates the possibility of exchange reactions. The frameworks are comprised of edge-sharing UO*_n_* and SiO_4_ polyhedra. Despite essential differences, these structures share some common features, i.e., the polysilicate anions in **1** and **2** are comprised of hexameric [Si_6_O_18_]^12−^ rings, while in **3** and **4**, of tetrameric [Si_4_O_12_]^8−^ rings. Topological analysis of other known uranyl silicate structures shows that these hexa- and tetrameric rings are found very commonly as secondary building blocks.

## Figures and Tables

**Table 1:** Parameters of the experiment and structure refinement for Rb_2_[(UO_2_)_2_(Si_8_O_19_)](H_2_O)_2.5_ (**1**), (K,Rb)_2_[(UO_2_)(Si_10_O_22_)] (**2**), [Rb_3_Cl][(UO_2_)(Si_4_O_10_)] (**3**), and [Cs_3_Cl][(UO_2_)(Si_4_O_10_)] (**4**).

	1	2	3	4
	**Crystal Data**			
Temperature (K)	150			
Radiation	MoKα, 0.71073			
Crystal system	orthorhombic	monoclinic	orthorhombic	orthorhombic
Space group	*C* *mce*	*C*2/*m*	*I* *mma*	*I* *mma*
*a* (Å)	14.5795(2)	23.0027(8)	15.2712(12)	15.4148(8)
*b* (Å)	14.2083(2)	8.0983(3)	7.9647(8)	7.9229(4)
*c* (Å)	23.1412(4)	11.9736(4)	12.4607(9)	13.0214(7)
β (°)		90.372(3)		
Volume (Å^3^)/Z	4793.70(13)/2	2230.43(14)/2	1515.6(2)/2	1590.30(14)/2
*D_calc_* (g/cm^3^)	3.546	2.972	3.656	4.079
*μ* (mm^−1^)	18.042	9.023	20.826	17.493
Crystal size (mm)	0.07 × 0.12 × 0.09	0.012 × 0.08 × 0.04	0.006 × 0.10 × 0.05	0.014 × 0.12 × 0.08
	**Data collection:**			
*θ* range (°)	3.303–28.000	3.403–29.262	2.109–27.994	4.007–27.935
*h*, *k*, *l* ranges	–18→19, −18→18, −30→30	–27→29, −10→9, −15→15	–20→19, −3→10, −14→16	–17→16, −9→7, −15→13
Total reflections collected	3007	2639	1005	890
Unique reflections (*R_int_*)	2703(0.018)	2197(0.033)	862(0.026)	846(0.024)
	**Structure refinement:**			
*R*_1_[*F* > 4*σF*], *wR*_1_[*F* > 4*σF*]	0.020, 0.067	0.026, 0.062	0.026, 0.055	0.020, 0.050
R_all_, wR_all_	0.023, 0.069	0.034, 0.064	0.035, 0.058	0.022, 0.051
Goodness-of-fit	1.056	1.072	1.080	1.161
CCDC number	2222962	2222964	2222965	2222966

## Data Availability

The crystal structures data are available at CCDC by number: 2222962, 2222964, 2222965, 2222966.

## Supplementary Materials

The following supporting information can be downloaded at: https://www.mdpi.com/article/10.3390/ma16114153/s1, Table S1. The types of Si_n_O_m_ complexes in the structures of uranyl silicate; Table S2. Selected interatomic bonds in the structure of Rb_2_[(UO_2_)_2_(Si_8_O_19_)](H_2_O)_2.5_; Table S3. Selected interatomic bonds in the structure of (K,Rb)_2_[(UO_2_)(Si_10_O_22_)]; Table S4. Selected interatomic bonds in the structure of [Rb_3_Cl][(UO_2_)(Si_4_O_10_)]; Table S5. Selected interatomic bonds in the structure of [Cs_3_Cl][(UO_2_)(Si_4_O_10_)]; Figure S1. Powder XRD of (K,Rb)_2_[(UO_2_)(Si_10_O_22_)] and Rb_2_[(UO_2_)_2_(Si_8_O_19_)](H_2_O)_2.5_; Figure S2. Powder XRD of [Rb_3_Cl][(UO_2_)(Si_4_O_10_)] and [Cs_3_Cl][(UO_2_)(Si_4_O_10_)]; Figure S3. IR absorption spectra of (K,Rb)_2_[(UO_2_)(Si_10_O_22_)], Rb_2_[(UO_2_)_2_(Si_8_O_19_)](H_2_O)_2.5_, [Rb_3_Cl][(UO_2_)(Si_4_O_10_)] and [Cs_3_Cl][(UO_2_)(Si_4_O_10_)]; Table S6. Chemical data (in wt %) for (K,Rb)_2_[(UO_2_)(Si_10_O_22_)]; Table S7. Chemical data (in wt %) for Rb_2_[(UO_2_)_2_(Si_8_O_19_)](H_2_O)_2.5_; Table S8. Chemical data (in wt %) for [Rb_3_Cl][(UO_2_)(Si_4_O_10_)]; Table S9. Chemical data (in wt %) for [Cs_3_Cl][(UO_2_)(Si_4_O_10_)]; Table S10. Bond valences parameters in structure of Rb_2_[(UO_2_)_2_(Si_8_O_19_)](H_2_O)_2.5_; Table S11. The BVS parameters for Si-O bonds in the structure of uranyl silicates; Table S12. Bond valences parameters in structure of (K,Rb)_2_[(UO_2_)(Si_10_O_22_)]; Table S13. Bond valences parameters in structure of [Rb_3_Cl][(UO_2_)(Si_4_O_10_)]; Table S14. Bond valences parameters in structure of [Cs_3_Cl][(UO_2_)(Si_4_O_10_)]. References [57,58,59,60,61,62,63] are cited in the supplementary materials.

## References

[1] Belova L.N., Doynikova O.A. (2003). Formation conditions of uranium minerals in oxidation zone of uranium deposits. Geol. Ore Deposit..

[2] Finch R.J., Buck E.C., Finn P.A., Bates J.K. (1999). Oxidative corrosion of spent UO_2_ fuel in vapor and dripping groundwater at 90 °C. MRS Online Proc. Libr..

[3] Plášil J. (2018). Mineralogy, crystallography and structural complexity of natural uranyl silicates. Minerals.

[4] Burns P.C. (1998). The Structure of Boltwoodite and Implications of Solid Solution toward Sodium Boltwoodite. Can. Mineral..

[5] Zolotarev A.A., Krivovichev S.V., Avdontseva M.S. (2011). Cs-exchanged cuprosklodowskite. Miner. Adv. Mater..

[6] Wronkiewicz D.J., Bates J.K., Wolf S.F., Bick E.C. (1996). Ten-year results from unsaturated drip tests with UO_2_ at 90 °C: Implications for the corrosion of spent nuclear fuel. J. Nucl. Mater..

[7] Burns P.C., Olson R.A., Finch R.J., Hanchar J.M., Thibault Y. (2000). KNa_3_(UO_2_)_2_(Si_4_O_10_)_2_(H_2_O)_4_, a new compound formed during vapor hydration of an actinide-bearing borosilicate waste glass. J. Nucl. Mater..

[8] Burns P.C. (2005). U^6+^ Minerals and Inorganic Compounds: Insights into an Expanded Structural Hierarchy of Crystal Structures. Can. Mineral..

[9] Plášil J., Fejfarová K., Čejka J., Dušek M., Škoda R., Sejkora J. (2013). Revision of the crystal structure and chemical formula of haiweeite, Ca(UO_2_)_2_(Si_5_O_12_)(OH)_2_ 6H_2_O. Am. Mineral..

[10] Chernikov A.A., Sidorenko G.A., Valueva A.A. (1977). New data on uranyl minerals of the ursilite-weeksite group. Zap. Vseross. Mineral. Ova..

[11] Chernikov A.A., Krutetskaya O.V., Sidelnikova V.D. (1957). Ursilite—A new silicate of uranium. Voprosy Geol. Urana.

[12] Chen C.S., Kao H.M., Lii K.H. (2005). K_5_(UO_2_)_2_[Si_4_O_12_(OH)]: A uranyl silicate containing chains of four silicate tetrahedra linked by SiO···HOSi hydrogen bonds. Inorg. Chem..

[13] Morrison G., Smith M.D., Tran T.T., Halasyamani P.S., zur Loye H.C. (2015). Synthesis and structure of thenew pentanary uranium(vi) silicate, K_4_CaUSi_4_O_14_, a member of a structural family related to fresnoite. CrystEngComm.

[14] Morrison G., zur Loye H.C. (2016). Flux growth of [NaK_6_F][(UO_2_)_3_(Si_2_O_7_)_2_] and [KK_6_Cl][(UO_2_)_3_(Si_2_O_7_)_2_]: The effect of surface area to volume ratios on reaction products. Cryst. Growth Des..

[15] Wang X., Huang J., Liu L., Jacobson A.J. (2002). The novel open-framework uranium silicates Na_2_(UO_2_)(Si_4_O_10_) 2.1(H_2_O) (USH-1) and RbNa(UO_2_)(Si_2_O_6_)(H_2_O) (USH-3). J. Mater. Chem..

[16] Liu H.K., Lii K.H. (2011). Cs_2_USi_6_O_15_: A tetravalent uranium silicate. Inorg. Chem..

[17] Chen C., Chiang R., Kao H., Lii K. (2005). High-temperature, high-pressure hydrothermal synthesis, crystal structure, and solid-state NMR spectroscopy of Cs_2_(UO_2_)(Si_2_O_6_) and variable-temperature powder X-ray diffraction study of the hydrate phase Cs_2_(UO_2_)(Si_2_O_6_)·0.5H_2_O. Inorg. Chem..

[18] Huang J., Wang X., Jacobson A.J. (2003). Hydrothermal synthesis and structures of the new open-framework uranyl silicates Rb_4_(UO_2_)_2_(Si_8_O_20_) (USH-2Rb), Rb_2_(UO_2_)(Si_2_O_6_)H_2_O (USH-4Rb) and *A*_2_(UO_2_)(Si_2_O_6_)·0.5H_2_O (USH-5A.; *A* = Rb, Cs). J. Mater. Chem..

[19] Li H., Kegler P., Klepov V.V., Klinkenberg M., Bosbach D., Alekseev E.V. (2018). Comparison of uranium(VI) and thorium(IV) silicates synthesized via mixed fluxes techniques. Inorg. Chem..

[20] Plasil J. (2018). Structural complexity of uranophane and uranophane-β: Implications for their formation and occurrence. Eur. J. Miner..

[21] Li H., Langer E.M., Kegler P., Alekseev E.V. (2019). Structural and spectroscopic investigation of novel 2D and 3D uranium oxo-. silicates/germanates and some statistical aspects of uranyl coordination in oxo-salts. Inorg. Chem..

[22] Fejfarova K., Plasil J., Yang H., Cejka J., Dusek M., Downs R.T., Barkley M.C., Skoda R. (2012). Revision of the crystal structure and chemical formula of weeksite, K_2_(UO_2_)_2_(Si_5_O_13_)×4H_2_O. Am. Mineral..

[23] Liu H., Chang W., Lii K. (2011). High-temperature, high-pressure hydrothermal synthesis and characterization of an open-framework uranyl silicate with nine-ring channels: Cs_2_UO_2_Si_10_O_22_. Inorg. Chem..

[24] Babo J.M., Albrecht-Schmitt T.E. (2013). High temperature synthesis of two open-framework uranyl silicates with ten-ring channels: Cs_2_(UO_2_)_2_Si_8_O_19_ and Rb_2_(UO_2_)_2_Si_5_O_13_. J. Solid State Chem..

[25] Krivovichev S.V., Cahill C.L., Nazarchuk E.V., Armbruster T., Depmeier W. (2005). Chiral open-framework uranyl molybdates. 1. Topological diversity: Synthesis and crystal structure of [(C_2_H_5_)_2_NH_2_]_2_[(UO_2_)_4_(MoO_4_)_5_(H_2_O)](H_2_O). Micropor. Mesopor. Mater..

[26] Krivovichev S.V., Burns P.C., Armbruster T., Nazarchuk E.V., Depmeier W. (2005). Chiral open-framework uranyl molybdates. 2. Flexibility of the U:Mo = 6:7 frameworks: Syntheses and crystal structures of (UO_2_)_0.82_[C_8_H_20_N]_0.36_[(UO_2_)_6_(MoO_4_)_7_(H_2_O)_2_](H_2_O)_n_ and [C_6_H_14_N_2_][(UO_2_)_6_(MoO_4_)_7_(H_2_O)_2_](H_2_O)_m_. Micropor. Mesopor. Mater..

[27] Krivovichev S.V., Armbruster T., Chernyshov D.Y., Burns P.C., Nazarchuk E.V., Depmeier W. (2005). Chiral open-framework uranyl molybdates. 3. Synthesis, structure and the *C*222_1_ → *P*2_1_2_1_2_1_ low-temperature phase transition of [C_6_H_16_N]_2_[(UO_2_)_6_(MoO_4_)_7_(H_2_O)_2_](H_2_O)_2_. Micropor. Mesopor. Mater..

[28] Nazarchuk E.V., Krivovichev S.V., Burns P.C. (2005). Crystal structure and phase transformations of Ca[(UO_2_)_6_(MoO_4_)_7_(H_2_O)_2_](H_2_O)_n_ (n ~ 7.6). Zap. Vseross. Mineral. Ova..

[29] Nazarchuk E.V., Krivovichev S.V., Burns P.C. (2005). Crystal structure of Tl_2_[(UO_2_)_2_(MoO_4_)_3_] and crystal chemistry of the compounds *M*_2_[(UO_2_)_2_(MoO_4_)_3_] (*M* = Tl, Rb, Cs). Radiochemistry.

[30] Siidra O.I., Nazarchuk E.V., Charkin D.O., Ikhalaynen Y.A., Sharikov M.I. (2019). Open-framework sodium uranyl selenate and sodium uranyl sulfate with protonated morpholino-n-acetic acid. Z. Krist. Cryst. Mater..

[31] Siidra O.I., Nazarchuk E.V., Bocharov S.N., Depmeier W., Kayukov R.A. (2017). Microporous uranyl chromates successively formed by evaporation from acidic solution. Z. Krist. Cryst. Mater..

[32] Jouffret L., Rivenet M., Abraham F. (2010). A new series of pillared uranyl-vanadates based on uranophane-type sheets in the uranium-vanadium-linear alkyl diamine systems. J. Solid State Chem..

[33] Jouffret L., Shao Z., Rivenet M., Abraham F. (2010). New three-dimensional inorganic frameworks based on the uranophane-type sheet in monoamine templated uranyl-vanadates. J. Solid State Chem..

[34] Doran M.B., Stuart C.L., Norquist A.J., O’Hare D. (2004). [N_2_C_6_H_14_]_2_[(UO_2_)_6_(H_2_O)_2_F_2_(PO_4_)_2_(HPO_4_)_4_]·4H_2_O:  A New Microporous Uranium Phosphate Fluoride. Chem. Mater..

[35] Danis J.A., Runde W.H., Scott B., Fettinger J., Eichhorn B. (2001). Hydrothermal synthesis of the first organically templated open-framework uranium phosphate. Chem. Commun..

[36] Doran M.B., Norquist A.J., O’Hare D. (2002). [NC_4_H_12_]_2_[(UO_2_)_6_(H_2_O)_2_(SO_4_)_7_]: The first organically templated actinide sulfate with a three-dimensional framework structure. Chem. Commun..

[37] Alekseev E.V., Krivovichev S.V., Depmeier W. (2008). A Crown ether as template for microporous and nanostructured uranium compounds. Angew. Chem. Int. Ed. Engl..

[38] Bharara M.S., Gorden A.E.V. (2010). Amine templated two- and three-dimensional uranyl sulfates. Dalton Trans..

[39] Yang W., Parker T.G., Sun Z.M. (2015). Structural chemistry of uranium phosphonates. Coord. Chem. Rev..

[40] Juillerat C.A., Moore E.E., Morrison G., Smith M.D., Besmann T., Zur Loye H.C. (2018). Versatile uranyl germanate framework hosting 12 different alkali halide 1D salt inclusions. Inorg. Chem..

[41] Dal Bo F.D., Aksenov S.M., Burns P.C. (2019). A novel family of microporous uranyl germanates: Framework topology and complexity of the crystal structures. J. Solid State Chem..

[42] Liu H.K., Peng C.C., Chang W.J., Lii K.H. (2016). Tubular chains, single layers, and multiple chains in uranyl silicates: *A*_2_[(UO_2_)Si_4_O_10_] (*A* = Na, K, Rb, Cs). Cryst. Growth Des..

[43] Liu C., Liu H., Chang W., Lii K. (2015). K_2_Ca_4_[(UO_2_)(Si_2_O_7_)_2_]: A uranyl silicate with a one-dimensional chain structure. Inorg. Chem..

[44] Lee C., Wang S., Chen Y., Lii K. (2009). Flux synthesis of salt-inclusion uranyl silicates: [K_3_Cs_4_F][(UO_2_)_3_(Si_2_O_7_)_2_] and [NaRb_6_F][(UO_2_)_3_(Si_2_O_7_)_2_]. Inorg. Chem..

[45] Farrugia L.J. (1999). WinGX suite for small-molecule single-crystal crystallography. J. Appl. Crystallogr..

[46] Dolomanov O.V., Bourhis L.J., Gildea R.J., Howard J.A.K., Puschmann H. (2009). OLEX2: A complete structure solution, refinement and analysis program. J. Appl. Cryst..

[47] Brown I.D. (2009). Recent developments in the methods and applications of the bond valence model. Chem. Rev..

[48] Brown I.D. (2002). The Chemical Bond in Inorganic Chemistry: The Bond Valence Model.

[49] Gagne O.C., Hawthorne F.C. (2016). Bond-length distributions for ions bonded to oxygen: Results for the transition metals and quantification of the factors underlying bond-length variation in inorganic solids. Acta Crystallogr. Sect. B Struct. Sci..

[50] Heinrich A., Gramlich V. (1982). Cs_2_Cu_2_Si_8_O_19_, a new double layer silicate structure. Naturwissenschaften.

[51] Watanabe I., Kawahara A. (1993). Structure of a synthetic double-layer silicate, Rb_2_Cu_2_Si_8_O_19_. Acta Crystallogr. Sect. C Cryst. Struct. Commun..

[52] Krueger H., Kahlenberg V., Kaindl R. (2005). Structural studies on Na_6_Si_8_O_19_-a monophyllosilicate with a new type of layered silicate anion. Solid State Sci..

[53] Morrison G., Tran T.T., Halasyamani P.S., zur Loye H.C. (2016). K_8_(K_5_F)U_6_Si_8_O_40_: An intergrowth uranyl silicate. Inorg. Chem..

[54] Blaton N., Vochten R., Peters O.M., van Springel K. (1999). The crystal structure of Na_2_(UO_2_)_2_SiO_4_F_2_, a compound structurally related to soddyite, and formed during uranyl silicate synthesis in Teflon-lined bombs. Neues Jahrb. Mineral Abh..

[55] Galli E., Gottardi G., Pongiluppi D. (1979). The crystal structure of the zeolite merlinoite. Neues Jahrb. Mineral. Abh..

[56] Pakhomova A.S., Armbruster T., Krivovichev S.V., Yakovenchuk V.N. (2014). Dehydration of the zeolite merlinoite from the Khibiny massif, Russia: An in situ temperature-dependent single-crystal X-ray study. Eur. J Mineral..

[57] Kobatko K.A., Burns P.C. (2006). A novel arrangement of silicate tetrahedra in the uranyl sheet of oursinite, (Co_0.8_Mg_0.2_)[(UO_2_)(SiO_3_OH)]_2_(H_2_O)_6_. Amer. Mineral..

[58] Plášil J., Petříček V., Locock A.J., Škoda R., Burns P.C. (2017). The (3 + 3) commensurately modulated structure of the uranyl silicate mineral swamboite-(Nd), Nd_0.333_[(UO_2_)(SiO_3_OH)](H_2_O)_2.41_. Z. Kristallogr..

[59] Colmenero F., Bonales L.J., Cobos J., Timón V. (2017). Structural, mechanical and vibrational study of uranyl silicate mineral soddyite by DFT calculations. J. Solid State Chem..

[60] Chen Y.H., Liu H.K., Chang W.J., Tzou D.L., Lii K.H. (2016). High-temperature, high-pressure hydrothermal synthesis, characterization, and structural relationships of mixed-alkali metals uranyl silicates. J. Solid State Chem..

[61] Read C.M., Smith M.D., Withers R., zur Loye H.C. (2015). Flux crystal growth and optical properties of two uranium-containing silicates: *A*_2_USiO_6_ (*A* = Cs, Rb). Inorg. Chem..

[62] Plaisier J.R., IJdo D.J.W., de Mello Donega C., Blasse G. (1995). Structure and luminescence of barium uranium disilicate (BaUO_2_Si_2_O_6_). Chem. Mater..

[63] Morrison G., Smith M.D., zur Loye H.C. (2017). Flux versus hydrothermal growth: Polymorphism of *A*_2_(UO_2_)Si_2_O_6_ (*A* = Rb, Cs). Inorg. Chem..

