# Supplementary material for: Framework Uranyl Silicates: Crystal Chemistry and a New Route for the Synthesis
Source: Materials (Basel). 2023 Jun 2;16(11):4153. doi: 10.3390/ma16114153 (PMC10254168; doi:10.3390/ma16114153)
Supplement: Supplementary file 1 [file materials-16-04153-s001.zip › materials-2381421-supplementary.pdf]

Supporting Information

Framework uranyl silicates: crystal chemistry and a new route for the synthesis.

Evgeny V. Nazarchuk[1]*, Oleg I. Siidra[1,2], Dmitri O. Charkin[3] and Yana G. Tagirova[1]

[1] 1Department of Crystallography, Saint-Petersburg State University, University emb. 7/9, St. Petersburg 199034, Russia, e_nazarchuk@mail.ru
[2] Kola Science Center, Russian Academy of Sciences, Apatity, Murmansk Region, 184200, Russia, siidra@mail.ru
[3] Department of Chemistry, Moscow State University, Vorobievy Gory 1, bd. 3, Moscow 119991, Russia, d.o.charkin@gmail.com
***** Correspondence: e_nazarchuk@mail.ru

Table of contents

Section	Caption	Page
Table S1	The types of Si_nO_m complexes in the structures of uranyl silicate	3
Table S2	Selected interatomic bonds in the structure of $Rb_2[(UO_2)_2(Si_8O_{19})](H_2O)_{2.5}$	8
Table S3	Selected interatomic bonds in the structure of $(K,Rb)_2[(UO_2)(Si_{10}O_{22})]$	8
Table S4	Selected interatomic bonds in the structure of $[Rb_3Cl][(UO_2)(Si_4O_{10})]$	9
Table S5	Selected interatomic bonds in the structure of $[Cs_3Cl][(UO_2)(Si_4O_{10})]$	9
Figure S1	Powder XRD of $(K,Rb)_2[(UO_2)(Si_{10}O_{22})]$ and $Rb_2[(UO_2)_2(Si_8O_{19})](H_2O)_{2.5}$	10
Figure S2	Powder XRD of $[Rb_3Cl][(UO_2)(Si_4O_{10})]$ and $[Cs_3Cl][(UO_2)(Si_4O_{10})]$	11
Figure S3	IR absorption spectra of $(K,Rb)_2[(UO_2)(Si_{10}O_{22})]$, $Rb_2[(UO_2)_2(Si_8O_{19})](H_2O)_{2.5}$, $[Rb_3Cl][(UO_2)(Si_4O_{10})]$ and $[Cs_3Cl][(UO_2)(Si_4O_{10})]$	12
Table S6	Chemical data (in wt %) for $(K,Rb)_2[(UO_2)(Si_{10}O_{22})]$	13
Table S7	Chemical data (in wt %) for $Rb_2[(UO_2)_2(Si_8O_{19})](H_2O)_{2.5}$	13
Table S8	Chemical data (in wt %) for $[Rb_3Cl][(UO_2)(Si_4O_{10})]$	13
Table S9	Chemical data (in wt %) for $[Cs_3Cl][(UO_2)(Si_4O_{10})]$	14
Table S10	Bond valences parameters in structure of $Rb_2[(UO_2)_2(Si_8O_{19})](H_2O)_{2.5}$	14
Table S11	The BVS parameters for Si-O bonds in the structure of uranyl silicates	15
Table S12	Bond valences parameters in structure of $(K,Rb)_2[(UO_2)(Si_{10}O_{22})]$	17
Table S13	Bond valences parameters in structure of $[Rb_3Cl][(UO_2)(Si_4O_{10})]$	17
Table S14	Bond valences parameters in structure of $[Cs_3Cl][(UO_2)(Si_4O_{10})]$	17

Table S1. The types of Si_nO_m complexes in the structures of uranyl silicate.

Complex type	Dimensions	Chemical formula	Space group	*a*, Å / *α*,º	*b*, Å / *β*,º	*c*, Å / *γ*, º	Ref.
**$[SiO_4]^{4-}$**	0D	α-uranophane, $Ca[(UO_2)_2(SiO_3OH)]_2(H_2O)_5$	*P*2$_1$	15.909	7.002 / 97.27	6.665	[1]
		β-uranophane, $Ca[(UO_2)_2(SiO_3OH)]_2(H_2O)_5$	*P*2$_1$/*a*	13.966	15.443 / 91.38	6.632	[1]
		kasolite, $Pb[(UO_2)(SiO_4)](H_2O)$	*P*2$_1$/*c*	6.70	6.93 / 105.00	13.28	[2]
		boltwoodite, $K[(UO_2)(SiO_3OH)]_2(H_2O)_{1.5}$	*P*2$_1$/*m*	7.077	7.060 / 104.98	6.648	[3]
		natroboltwoodite, $Na[(UO_2)(SiO_3OH)](H_2O)$	*P*2$_1$2$_1$2$_1$	27.40	7.02	6.65	[3]
		sklodowskite, $Mg[(UO_2)(SiO_3OH)]_2(H_2O)_6$	*C*2/*m*	17.382	7.047 / 105.88	6.610	[2]
		cuprosklodowskite, $Cu[(UO_2)(SiO_3OH)]_2(H_2O)_6$	*P*-1	7.052 / 109.23	9.267 / 89.84	6.655 / 110.01	[2]
		oursinite, $Co[(UO_2)(SiO_3OH)]_2(H_2O)_6$	*Cmca*	7.04	17.55	12.73	[4]
		swamboite, $Nd_{0.333}[(UO_2)(SiO_3OH)](H_2O)_{2.41}$	*P*2$_1$/*a*	6.65	6.98 / 102.59	8.80	[5]
		soddyite, $[(UO_2)_2(SiO_4)](H_2O)_2$	*Fddd*	8.334	11.212	18.668	[6]
		$Na_2[(UO_2)_2(SiO_4)F_2]$	*I*4$_1$/*amd*	6.975	6.975	18.31	[7]
**$[Si_2O_7]^{6-}$**	0D	$Na_6[(UO_2)_3(Si_2O_7)_2]$	*C*2/*m*	22.323	7.463 / 99.377	5.777	[8]
		$[Na_3K_3][(UO_2)_3(Si_2O_7)_2](H_2O)_2$	*P*-1	5.798 / 103.59	7.588 / 102.88	12.807 / 90.06	[9]
		$[Na_3Rb_3][(UO_2)_3(Si_2O_7)_2]$	*P*-1	5.799 / 78.265	7.575 / 79.14	12.937 / 89.94	[9]
		$[K_3Cs_4F][(UO_2)_3(Si_2O_7)_2]$	*Cmc*2$_1$	7.809	22.282	14.086	[10]
		$[NaRb_6F][(UO_2)_3(Si_2O_7)_2]$	*Pnnm*	11.143	13.515	7.887	[10]
		$[NaK_6F][(UO_2)_3(Si_2O_7)_2]$	*Pnnm*	11.082	13.115	7.842	[11]
		$[KK_6Cl][(UO_2)_3(Si_2O_7)_2]$	*Pnnm*	11.083	13.585	7.869	[11]
		$[Cs_2Cs_5F][(UO_2)_3(Si_2O_7)_2]$	*P*2$_1$/*n*	7.556	9.864 / 91.71	18.554	[11]
		$[K_2Ca_4][(UO_2)(Si_2O_7)_2]$	*P*-1	6.635 / 98.32	6.679 / 93.62	9.699 / 112.31	[12]
		$[K_4Na_2][(UO_2)_3(Si_2O_7)_2](H_2O)_3$	*C*2/*m*	13.154	15.159 / 104.64	5.824	[12]
		$[K_8K_5F][U_6(Si_8O_{40})]$	*P*2$_1$2$_1$2$_1$	11.803	13.698	26.679	[11]
**$[Si_4O_{12}]^{8-}$**	0D	$Cs_2[USiO_6]$	*Immm*	8.581	13.001	13.881	[13]
		$Rb_2[USiO_6]$	*Immm*	8.492	12.668	13.508	[13]

		$Rb_2[(UO_2)(Si_2O_6)](H_2O)$	*P*2$_1$/*n*	7.699	20.97 / 97.92	12.05	[14]
		$[RbNa][(UO_2)(Si_2O_6)(H_2O)]$	*P*-1	7.367 / 78.02	7.869 / 75.01	8.177 / 83.74	[15]
		$[K_4Ca][U(Si_2O_7)_2]$	*P*-1	6.635 / 98.32	6.679 / 93.62	9.699 / 112.31	[12]
		$[K_8K_5F][U_6(Si_8O_{40})]$	*P*2$_1$2$_1$2$_1$	11.803	13.698	26.679	[16]
**$[Si_4O_{12}]^{8-}$**	0D	$[Cs_9Cs_6Cl][(UO_2)_7(Si_6O_{17})_2(Si_4O_{12})]$	*P*-1	7.350 / 89.29	15.332 / 89.99	17.255 / 76.45	[16]
**$[Si_4O_{12}(OH)]^{9-}$**	0D	$K_5[(UO_2)_2(Si_4O_{12}(OH))]$	*Pbcm*	13.127	12.264	22.233	[17]
**$[Si_2O_6]^{4-}$**	1D	$K_2[(UO_2)Si_2O_6]$	*C*2/*c*	21.695	14.416 / 136.71	16.073	[18]
		$Ba[(UO_2)(Si_2O_6)]$	*Cmcm*	5.703	16.464	7.496	[19]
		α-$Cs_2[(UO_2)(Si_2O_6)]$	*Ibca*	15.137	15.295	16.401	[17]
		$Rb_2[(UO_2)(Si_2O_6)](H_2O)_{0.5}$	*Pbca*	14.627	15.145	16.645	[14]
		$Cs_2[(UO_2)(Si_2O_6)](H_2O)_{0.5}$	Pbca	15.047	15.427	16.732	[14]
		$Rb_2[(UO_2)Si_2O_6]$	*I*2/*a*	14.993	14.803 / 90.72	16.238	[20]
		β-$Cs_2[(UO_2)(Si_2O_6)]$	*C*2	12.136	10.043 / 95.85	7.789	[20]
**$[Si_4O_{10}]^{4-}$**	1D	$[Cs_3F][(UO_2)(Si_4O_{10})]$	*Imma*	15.476	7.815	12.756	[16]
		$Na_2[(UO_2)(Si_4O_{10})](H_2O)_{0.5}$	*I*4/*mcm*	18.033	18.033	7.775	[21]
		$Cs_2[(UO_2)(Si_4O_{10})]$	*Cmca*	7.72	19.87	24.02	[21]
		$Rb_2[(UO_2)(Si_4O_{10})]$	*P*2$_1$/*c*	10.348	23.730 / 110.48	7.628	[21]
**$[Si_5O_{13}]^{6-}$**	1D	haiweeite, $Ca[(UO_2)_2(Si_5O_{12})(OH)_2](H_2O)_6$	*Pbcn*	18.30	14.23	17.92	[22]
**$[Si_6O_{17}]^{10-}$**	1D	$[Cs_2Cs_5F][(UO_2)_2(Si_6O_{17})]$	*P*2$_1$2$_1$2	10.374	19.280	7.180	[11]
		$[Cs_9Cs_6Cl][(UO_2)_7(Si_6O_{17})_2(Si_4O_{12})]$	*P*-1	7.350 / 89.29	15.332 / 89.99	17.255/ 76.45	[11]
**$[Si_8O_{20}]^{8-}$**	1D	$Rb_4[(UO_2)(Si_8O_{20})]$	*P*-1	6.844 / 72.79	8.314 / 88.74	11.273 / 77.95	[14]
**$[Si_{10}O_{30}]^{20-}$**	1D	$K_{14}[(UO_2)_3Si_{10}O_{30}]$	*P*2$_1$/*c*	12.902	11.125 / 90.80	13.952	[21]
**$[Si_4O_{10}]^{4-}$**	2D	$KNa_3[(UO_2)_2(Si_4O_{10})_2](H_2O)_4$	*C*2	12.782	13.654 / 119.24	8.268	[23]
		α-$K_2[(UO_2)Si_4O_{10}]$	*P*2$_1$/*n*	8.297	7.7403 / 104.83	8.7906	[21]
		β-$K_2[(UO_2)(Si_4O_{10})]$	*C*2/*c*	17.933	6.793 / 125.52	11.744	[8]
		$Na_4[(UO_2)(Si_4O_{10})_2](H_2O)_4$	*P*2$_1$/*n*	7.048	11.413 / 90.56	12.027	[2]

		$Na_2[(UO_2)(Si_4O_{10})](H_2O)_{2.1}$	*C2/m*	12.772	13.614 / 119.26	8.247	[15]
**$[Si_5O_{13}]^{6-}$**	2D	weeksite, $K_2[(UO_2)_2(Si_5O_{13})](H_2O)_4$	*C2/m*	14.19	14.22	9.63	[2]
		$Rb_2[(UO_2)Si_5O_{13}]$	*C*222	7.118	17.949	7.057	[24]
**$[Si_8O_{20}]^{8-}$**	2D	$K_4[(UO_2)_2(Si_8O_{20})](H_2O)_4$	*P*2₁	14.179	14.179	14.965	[8]
**$[Si_{10}O_{22}]^{4-}$**	2D	$Cs_2[(UO_2)Si_{10}O_{22}]$	*P*2₁/*c*	12.251	8.052 / 90.01	23.380	[25]

References

1. Plášil, J. Structural complexity of uranophane and uranophane-β: Implications for their formation and occurrence. *Eur. J. Mineral*, 2018, 30, 253-257. DOI: 10.1127/ejm/2017/0029-2691
2. Plášil, J. Mineralogy, crystallography and structural complexity of natural uranyl silicates. *Minerals*. **2018**, *8*, 551-565. DOI: 10.3390/min8120551
3. Burns, P.C. The structure of boltwoodite and implications of solid solution toward sodium boltwoodite. *Can. Mineral.* **1998**, *36*, 1069-1075. https://rruff.info/doclib/cm/vol36/CM36_1069.pdf
4. Kobatko, K. A., Burns, P. C. A novel arrangement of silicate tetrahedra in the uranyl sheet of oursinite, $(Co_{0.8}Mg_{0.2})[(UO_2)(SiO_3OH)]_2(H_2O)_6$. *Amer. Mineral*. **2006**, 91, 333-336.
5. Plášil, J, Petříček, V., Locock, A.J., Škoda R., Burns, P.C. (2017) The (3 + 3) commensurately modulated structure of the uranyl silicate mineral swamboite-(Nd), $Nd_{0.333}[(UO_2)(SiO_3OH)](H_2O)_{2.41}$. *Z. Kristallogr*. **2017**, 233, 223-232. DOI: 10.1515/zkri-2017-2119.
6. Colmenero, F., Bonales, L. J., Cobos, J., Timón, V. (2017) Structural, mechanical and vibrational study of uranyl silicate mineral soddyite by DFT calculations. *J. Solid State Chem*. **2017**, 253, 249-257.
7. Blaton, N., Vochten, R., Peters, O. M., van Springel, K. The crystal structure of $Na_2(UO_2)_2SiO_4F_2$, a compound structurally related to soddyite, and formed during uranyl silicate synthesis in Teflon-lined bombs. *Neues Jahrb Mineral Abh*. **1999**, 253-264
8. Li, H., Langer, E.M., Kegler, P., Alekseev, E.V. Structural and spectroscopic investigation of novel 2D and 3D uranium oxo-. silicates/germanates and some statistical aspects of uranyl coordination in oxo-salts. *Inorg. Chem*. **2019**, *58*, 10333-10345. DOI: 10.1021/acs.inorgchem.9b01523
9. Chen, Y.H.; Liu, H.K.; Chang, W.J.; Tzou, D.L.; Lii, K.H. High-temperature, high-pressure hydrothermal synthesis, characterization, and structural relationships of mixed-alkali metals uranyl silicates. *J. Solid State Chem*. **2016**, 236, 55–60.
10. Gagne, O.C. and Hawthorne, F.C. Bond-length distributions for ions bonded to oxygen: Results for the transition metals and quantification of the factors underlying bond-length variation in inorganic solids. *Acta Crystallogr, Sect B: Struct Sci*. **2016**, *72*, 602-625. DOI: 10.1107/S2052252520005928
11. Morrison, G. and zur Loye, H. C. Flux growth of $[NaK_6F][(UO_2)_3(Si_2O_7)_2]$ and $[KK_6Cl][(UO_2)_3(Si_2O_7)_2]$: The effect of surface area to volume ratios on reaction products. *Cryst. Growth Des*. **2016**, *16*, 1294-1299. DOI: 10.1021/acs.cgd.5b01408

12. Liu, C., Liu, H., Chang, W. and Lii, K. $K_2Ca_4[(UO_2)(Si_2O_7)_2]$: A uranyl silicate with a one-dimensional chain structure. *Inorg. Chem.* **2015**, *54*, 8165-8167. DOI: 10.1021/acs.inorgchem.5b01390
13. Read C. M., Smith M. D., Withers R., and zur Loye H. C. Flux crystal growth and optical properties of two uranium-containing silicates: A_2USiO_6 (A = Cs, Rb). *Inorg. Chem.* **2015**, 54, 9, 4520–4525. Doi: 10.1021/acs.inorgchem.5b00364
14. Huang, J., Wang, X., Jacobson, A.J. Hydrothermal synthesis and structures of the new open-framework uranyl silicates $Rb_4(UO_2)_2(Si_8O_{20})$ (USH-2Rb), $Rb_2(UO_2)(Si_2O_6)H_2O$ (USH-4Rb) and $A_2(UO_2)(Si_2O_6)\cdot 0.5H_2O$ (USH-5A; A = Rb, Cs). *J. Mater. Chem.* **2003**, *13*, 191-196. DOI: 10.1039/B208787C
15. Wang, X., Huang, J., Liu, L. and Jacobson, A. J. The novel open-framework uranium silicates $Na_2(UO_2)(Si_4O_{10})$ 2.1(H_2O) (USH-1) and $RbNa(UO_2)(Si_2O_6)(H_2O)$ (USH-3). *J. Mater. Chem.* **2002**, *12*, 406-410. DOI: 10.1039/B109878K
16. Morrison, G., Tran, T.T., Halasyamani, P.S., zur Loye, H.C. $K_8(K_5F)U_6Si_8O_{40}$: An intergrowth uranyl silicate. *Inorg. Chem.* **2016**, *55*, 3215-3217. DOI: 10.1021/acs.inorgchem.6b00242
17. Chen, C., Chiang, R., Kao, H., and Lii, K. High-temperature, high-pressure hydrothermal synthesis, crystal structure, and solid-state NMR spectroscopy of $Cs_2(UO_2)(Si_2O_6)$ and variable-temperature powder x-ray diffraction study of the hydrate phase $Cs_2(UO_2)(Si_2O_6)\cdot 0.5H_2O$. *Inorg. Chem.* **2005**, *44*, 3914-3918. DOI: 10.1021/ic0500199
18. Li, H., Kegler, P., Klepov, V.V., Klinkenberg, M., Bosbach, D., Alekseev, E.V. Comparison of uranium(VI) and thorium(IV) silicates synthesized via mixed fluxes techniques. *Inorg. Chem.* **2018**, *57*, 6734-6745. DOI: 10.1021/acs.inorgchem.8b01072
19. Plaisier J. R., IJdo D. J. W., de Mello Donega C., and Blasse G. Structure and luminescence of barium uranium disilicate ($BaUO_2Si_2O_6$). *Chem. Mater.* **1995**, 7, 4, 738–743. Doi: 10.1021/cm00052a020
20. Morrison G., Smith M. D., and zur Loye H. C. Flux versus hydrothermal growth: polymorphism of $A_2(UO_2)Si_2O_6$ (A = Rb, Cs). *Inorg. Chem.* **2017**, 56, 3, 1053–1056. Doi: 10.1021/acs.inorgchem.6b02931
21. Liu, H.K., Peng, C.C., Chang, W.J., and Lii, K.H. Tubular chains, single layers, and multiple chains in uranyl silicates: $A_2[(UO_2)Si_4O_{10}]$ (A = Na, K, Rb, Cs). *Cryst. Growth Des.* **2016**, *9*, 5268-5272. DOI: 10.1021/ACS.CGD.6B00817
22. Plášil, J., Fejfarová, K., Čejka, J., Dušek, M., Škoda, R., and Sejkora, J. Revision of the crystal structure and chemical formula of haiweeite, $Ca(UO_2)_2(Si_5O_{12})(OH)_2$ $6H_2O$. *Am. Mineral.* **2013**, *98*, 718-723. DOI: 10.2138/am.2013.4284
23. Burns, P.C., Olson, R.A., Finch, R.J., Hanchar, J.M., Thibault, Y. $KNa_3(UO_2)_2(Si_4O_{10})_2(H_2O)_4$, a new compound formed during vapor hydration of an actinide-bearing borosilicate waste glass. *J. Nucl. Mater.*, **2000**, *278*, 290-300. DOI:10.1016/S0022-3115(99)00247-0
24. Babo, J.M., Albrecht-Schmitt, T.E. High temperature synthesis of two open-framework uranyl silicates with ten-ring channels: $Cs_2(UO_2)_2Si_8O_{19}$ and $Rb_2(UO_2)_2Si_5O_{13}$. *J Solid State Chem.* **2013**, *197*, 186-190. DOI: 10.1016/j.jssc.2012.07.048
25. Liu, H.K., Lii, K.H. $Cs_2USi_6O_{15}$: A tetravalent uranium silicate. *Inorg. Chem.* **2011**, *50*, 5870-5872. DOI: 10.1021/ic200771p

Table. S2. Selected interatomic bonds in the structure of $Rb_2[(UO_2)_2(Si_8O_{19})](H_2O)_{2.5}$

U1-O2	1.796(4)	Rb1-OW2	2.582(14)	Rb2-OW1	2.907(6)
U1-O1	1.816(4)	Rb1-O1	2.943(3)	Rb2-O1	2.973(3)
**<*U-O$_{ap}$*>**	***1.806***	Rb1-O1	2.943(3)	Rb2-O1	2.973(3)
U1-O3	2.217(4)	Rb1-OW1	3.056(6)	Rb2-O10	3.001(4)
U1-O4	2.326(3)	Rb1-O5	3.115(3)	Rb2-OW3	3.059(8)
U1-O6	2.328(3)	Rb1-O5	3.115(3)	Rb2-OW3	3.059(8)
U1-O4	2.481(3)	Rb1-O9	3.125(4)	Rb2-O13	3.089(3)
U1-O6	2.499(3)	Rb1-O2	3.257(3)	Rb2-O13	3.089(3)
**<*U-O$_{eq}$*>**	***2.370***	Rb1-O2	3.257(3)	Rb2-O4	3.477(3)
Si1-O8	1.5946(10)	Rb1-OW3	3.376(8)	Rb2-O4	3.477(3)
Si1-O3	1.595(4)	Rb1-OW3	3.376(8)	Rb2-O1	3.548(3)
Si1-O11	1.619(3)	Rb1-O6	3.660(3)	Rb2-O1	3.548(3)
Si1-O12	1.626(3)	**<*Rb1-O*>**	***2.908***	**<*Rb2-O*>**	***2.938***
**<*Si1-O*>**	***1.609***				
Si2-O6	1.611(3)	Si3-O12	1.588(3)	Si4-O11	1.588(3)
Si2-O4	1.614(3)	Si3-O13	1.609(3)	Si4-O9	1.6075(13)
Si2-O5	1.620(3)	Si3-O10	1.6130(13)	Si4-O7	1.608(3)
Si2-O13	1.623(3)	Si3-O7	1.614(3)	Si4-O5	1.610(3)
**<*Si2-O*>**	**1.617**	**<*Si3-O*>**	**1.606**	**<*Si4-O*>**	**1.603**

Table. S3. Selected interatomic bonds in the structure of $(K,Rb)_2[(UO_2)(Si_{10}O_{22})]$

U1-O1	1.798(4)	U2-O2	1.794(4)	Si1-O11	1.587(3)
U1-O1	1.798(4)	U2-O2	1.794(4)	Si1-O13	1.610(3)
**<*U1-O$_{ap}$*>**	***1.798***	**<*U2-O$_{ap}$*>**	***1.794***	Si1-O7	1.610(3)
U1-O6	2.275(3)	U2-O5	2.269(3)	Si1-O4	1.6124(17)
U1-O6	2.275(3)	U2-O5	2.269(3)	**<*Si1-O*>**	***1.605***
U1-O6	2.275(3)	U2-O5	2.269(3)	Si2-O6	1.582(3)
U1-O6	2.275(3)	U2-O5	2.269(3)	Si2-O14	1.605(4)
**<*U1-O$_{eq}$*>**	***2.275***	**<*U2-O$_{eq}$*>**	***2.269***	Si2-O7	1.624(3)
				Si2-O10	1.6269(1)
Rb1-O8	3.015(4)	K2-O5	2.877(3)	**<*Si2-O*>**	***1.609***
Rb1-O1	3.064(5)	K2-O5	2.877(4)	Si3-O14	1.577(4)
Rb1-O6	3.114(3)	K2-O10	2.921(5)	Si3-O13	1.578(3)
Rb1-O6	3.114(3)	K2-O2	3.042(5)	Si3-O16	1.582(4)
Rb1-O9	3.410(3)	K2-O7	3.104(4)	Si3-O12	1.6025(1)
Rb1-O9	3.410(3)	K2-O7	3.104(4)	**<*Si3-O*>**	***1.585***
Rb1-O5	3.443(3)	K2-O6	3.294(3)	Si4-O16	1.573(4)
Rb1-O5	3.443(3)	K2-O6	3.294(3)	Si4-O15	1.5740(1)
Rb1-O12	3.484(5)	K2-O10	3.403(5)	Si4-O3	1.5905(16
Rb1-O8	3.510(5)	**<*K1-O*>**	***3.102***	Si4-O9	1.604(3)
**<*Rb1-O*>**	***3.301***			**<*Si4-O*>**	***1.585***
Si5-O5	1.586(3)				
Si5-O11	1.610(3)				
Si5-9	1.619(3)				
Si5-O8	1.6271(19)				
**<*Si5-O*>**	***1.611***				

Table. S4. Selected interatomic bonds in the structure of $[Rb_3Cl][(UO_2)(Si_4O_{10})]$

U1-O3	1.801(5)	Rb1-O1	3.053(4)	Rb2-O3	2.862(5)
U1-O3	1.801(5)	Rb1-O1	3.053(4)	Rb2-O5	3.244(6)
<U1-O_{ap}>	***1.801***	Rb1-O1	3.053(4)	Rb2-O5	3.244(5)
U1-O1	2.252(4)	Rb1-O1	3.053(4)	Rb2-O1	3.306(4)
U1-O1	2.252(4)	Rb1-Cl1	3.1256(7)	Rb2-O1	3.306(4)
U1-O1	2.252(4)	Rb1-Cl1	3.1256(7)	Rb2-Cl1	3.4010(8)
U1-O1	2.252(4)	Rb1-O2	3.164(6)	Rb2-Cl1	3.4010(8)
<U1-O_{eq}>	***2.252***	Rb1-O2	3.164(6)	***<Rb2-O>***	***3.252***
Si1-O1	1.583(4)	Rb1-O3	3.300(5)		
Si1-O4	1.6085(19)	Rb1-O3	3.300(5)		
Si1-O2	1.6085(15)	***<Rb1-O>***	***3.139***		
Si1-O5	1.620(2)				
<Si1-O>	***1.605***				

Table. S5. Selected interatomic bonds in the structure of $[Cs_3Cl][(UO_2)(Si_4O_{10})]$

U1-O3	1.805(4)	RB1-O2	3.146(5)	RB2-O3	3.089(4)
U1-O3	1.805(4)	RB1-O2	3.146(5)	RB2-O5	3.355(5)
<U1-O_{ap}>	***1.805***	RB1-O1	3.192(3)	RB2-O1	3.433(3)
U1-O1	2.261(3)	RB1-O1	3.192(3)	RB2-O1	3.433(3)
U1-O1	2.261(3)	RB1-O1	3.192(3)	RB2-O5	3.476(5)
U1-O1	2.261(3)	RB1-O1	3.192(3)	RB2-Cl1	3.5025(4)
U1-O1	2.261(3)	RB1-O3	3.209(4)	RB2-Cl1	3.5025(4)
<U1-O_{eq}>	***2.261***	RB1-O3	3.209(4)	***<Rb2-O>***	***3.398***
Si1-O1	1.585(3)	RB1-Cl1	3.2120(5)		
Si1-O5	1.6139(15)	RB1-Cl1	3.2120(5)		
Si1-O2	1.6190(14)	***<Rb1-O>***	***3.190***		
Si1-O4	1.6191(17)				
<Si1-O>	***1.609***				

Figure S1. Powder XRD of $(K,Rb)_2[(UO_2)(Si_{10}O_{22})]$ and $Rb_2[(UO_2)_2(Si_8O_{19})](H_2O)_{2.5}$

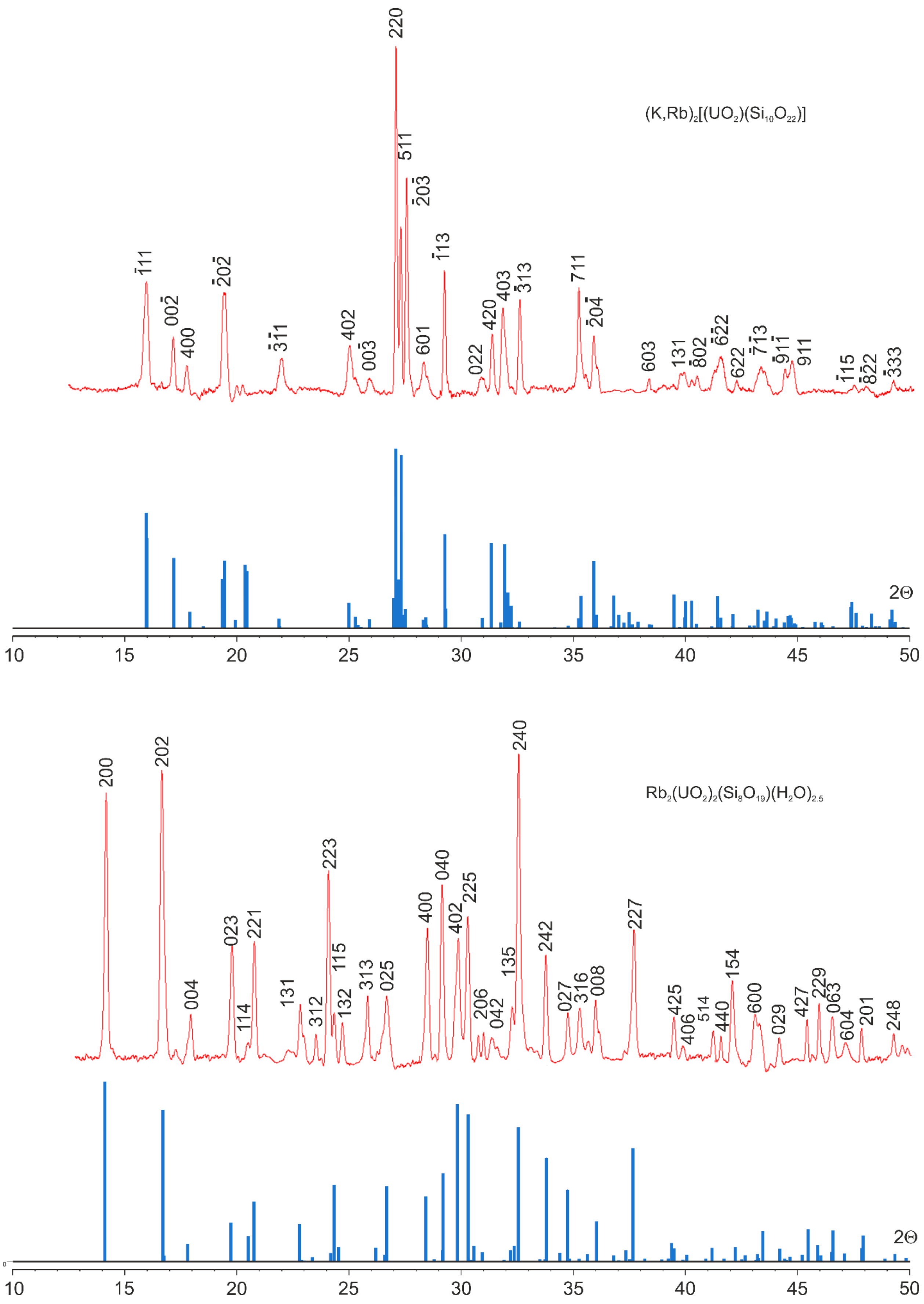

Figure S2. Powder XRD of $[Rb_3Cl][(UO_2)(Si_4O_{10})]$ and $[Cs_3Cl][(UO_2)(Si_4O_{10})]$

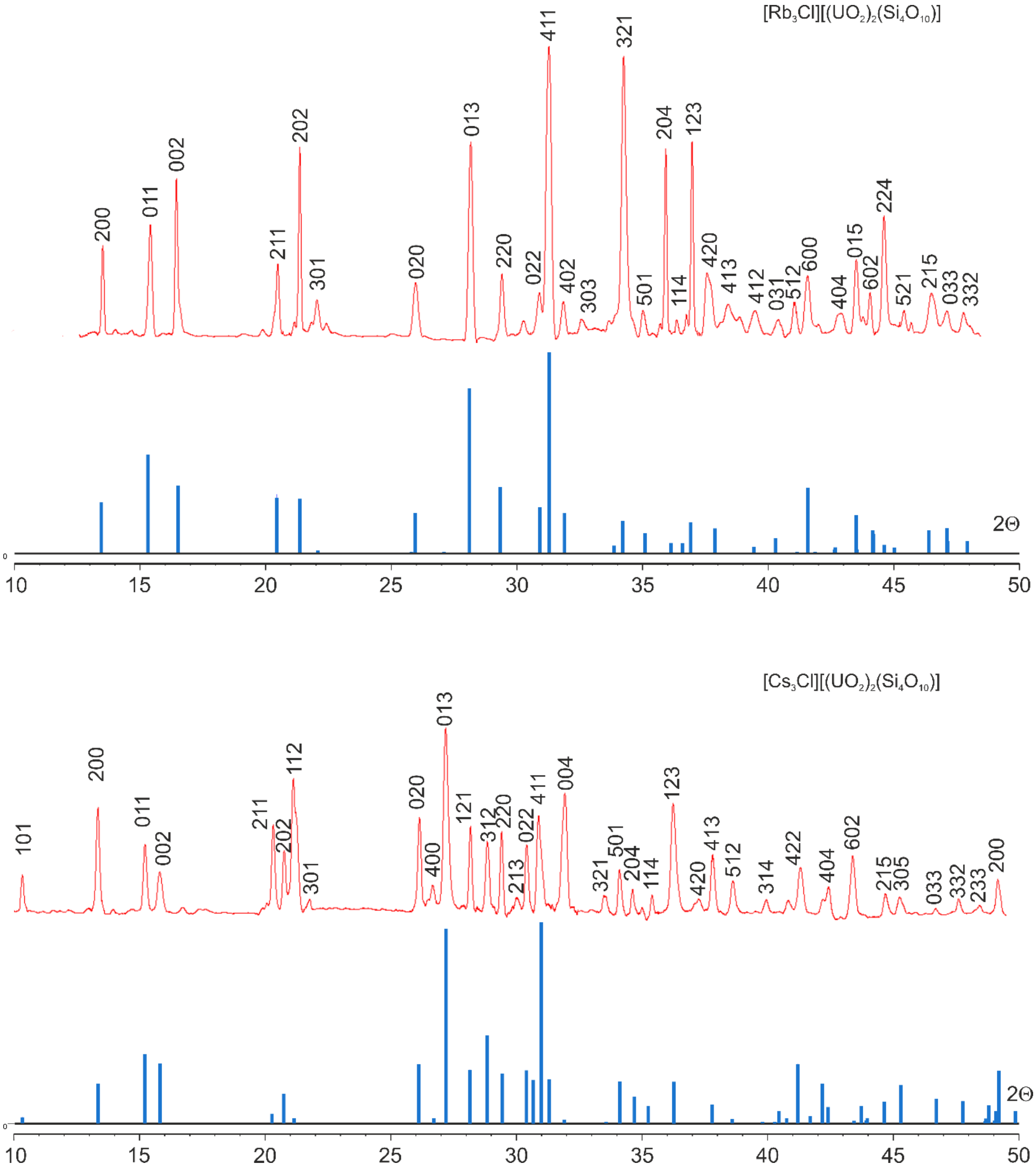

Figure S3. IR absorption spectra of $Rb_2[(UO_2)_2(Si_8O_{19})](H_2O)_{2.5}$ (a), $(K,Rb)_2[(UO_2)(Si_{10}O_{22})]$ (b), $[Rb_3Cl][(UO_2)(Si_4O_{10})]$ (c) and $[Cs_3Cl][(UO_2)(Si_4O_{10})]$ (d)

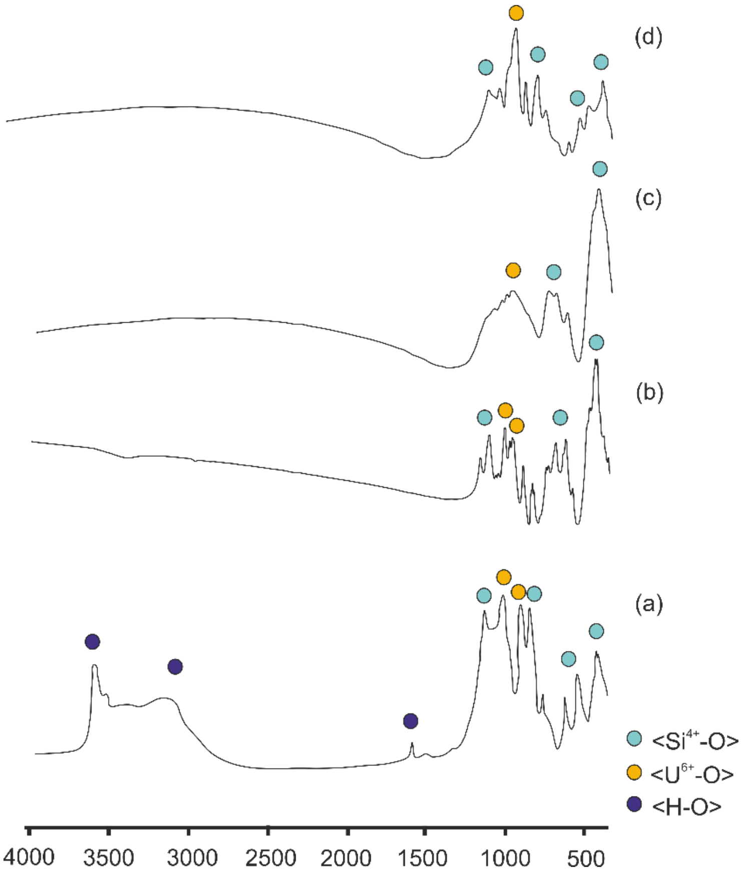

Table S6. Chemical data (in wt %) for $(K,Rb)_2[(UO_2)(Si_{10}O_{22})]$

Constituent	Mean	Range	Stand. Dev. (2σ)	Reference Material
UO_3	28,67	28.57 – 28.77	0.10	UO_2
SiO_2	61,93	61.80 – 62.06	0.13	$CaSiO_3$
Rb_2O	3,29	3.10 – 3.48	0.19	RbCl
K_2O	7.98	7.79 –8.17	0.19	KCl
**Total**	**101.87**			

The empirical formula calculated on the basis of 24 oxygen atoms per formula unit is $(Rb_{1,65}K_{0,34})[(U_{0,98}O_2)(Si_{10.04}O_{22})]$

Table S7. Chemical data (in wt %) for $Rb_2[(UO_2)_2(Si_8O_{19})](H_2O)_{2.5}$

Constituent	Mean	Range	Stand. Dev. (2σ)	Reference Material
UO_3	44,48	44.38 – 44.58	0.10	UO_2
SiO_2	37,37	37.24 – 37.50	0.13	$CaSiO_3$
Rb_2O	14,61	14.42 – 14.80	0.19	RbCl
H_2O	3,52	3.33 –3.71	0.19	
**Total**	**99.99**			

The empirical formula calculated on the basis of 25.5 oxygen atoms per formula unit is $Rb_{2,01}[(UO_2)_2(Si_{7,99}O_{19})](H_2O)_{2.52}$

Table S8. Chemical data (in wt %) for $[Rb_3Cl][(UO_2)(Si_4O_{10})]$

Constituent	Mean	Range	Stand. Dev. (2σ)	Reference Material
UO_3	33,58	33.48 – 33.68	0.10	UO_2
SiO_2	28,58	28.45 – 28.71	0.13	$CaSiO_3$
Rb_2O	33,59	33.40 – 33.68	0.19	RbCl
Cl	4,25	4.06 –4.44	0.19	Pyromorphite
O=Cl2	0,96			
**Total**	**99.03**			

The empirical formula calculated on the basis of 13 oxygen atoms per formula unit is $[Rb_{3.02}Cl][(U_{0.99}O_2)(Si_{4.01}O_{10})]$

Table S9. Chemical data (in wt %) for $[Cs_3Cl][(UO_2)(Si_4O_{10})]$

Constituent	Mean	Range	Stand. Dev. (2σ)	Reference Material
UO_3	29,29	29.19 – 29.39	0.10	UO_2
SiO_2	24,25	24.12 – 24.38	0.13	$CaSiO_3$
Cs_2O	42,86	42.69 – 43.03	0.17	CsCl
Cl	3,60	3.41 –3.79	0.19	Pyromorphite
O=Cl2	0,814			
**Total**	**99.19**			

The empirical formula calculated on the basis of 13 oxygen atoms per formula unit is $[Cs_3Cl][(U_{1.01}O_2)(Si_{3.98}O_{10})]$

Table S10. Bond valences parameters in structure of $Rb_2[(UO_2)_2(Si_8O_{19})](H_2O)_{2.5}$.

	**U1**	**Si1**	**Si2**	**Si3**	**Si4**	**Rb1**	**Rb2**	∑
**O1**	1.63					0.13×2	0.13×2<br>0.04×2	**1.93**
**O2**	1.70					0.07×2		**1.77**
**O3**	0.70	1.08						**1.78**
**O4**	0.55<br>0.4		1.03				0.04×2	**2.02**
**O5**			1.01		1.04	0.09×2		**2.14**
**O6**	0.55<br>0.38		1.03			0.03		**1.99**
**O7**				1.03	1.04			**2.07**
**O8**		1.08						**2.16**
**O9**					1.04	0.09		**2.17**
**O10**				1.03			0.12	**2.18**
**O11**		1.01			1.10			**2.11**
**O12**		0.99		1.10				**2.13**
**O13**			1.00	1.04			0.1×2	**2.14**
**OW1**						0.12	0.14	**0.26**
**OW2*[1]**						0.34		**0.34**
**OW3*[2]**						0.05	0.11×2	**0.16**
∑	**5.91**	**4.16**	**4.07**	**4.19**	**4.22**	**1.28**	**1.08**	

*[1] OW1 = 0.40, *[2] OW1 = 0.55

Table. S11. The BVS parameters for Si-O bonds in the structure of uranyl silicates.

compound	site	<Si-O>	S_{ij}
$K_{14}(UO_2)_3Si_{10}O_{30}$	Si1	1.628	3.97
	Si2	1.620	4.05
	Si3	1.625	3.99
	Si4	1.628	3.97
	Si5	1.632	3.93
$K_2(UO_2)Si_2O_6$	Si1	1.604	4.22
	Si2	1.611	4.14
	Si3	1.614	4.10
	Si4	1.612	4.13
β-$K_2(UO_2)Si_4O_{10}$	Si1	1.588	4.39
	Si2	1.596	4.31
$K_4(UO_2)_2Si_8O_{20}(H_2O)_4$	Si1	1.628	4.04
	Si2	1.577	4.53
	Si3	1.594	4.37
	Si4	1.653	3.75
	Si5	1.612	4.19
	Si6	1.591	4.36
	Si7	1.641	3.90
	Si8	1.610	4.17
	Si9	1.579	4.49
	Si10	1.629	4.01
	Si11	1.634	3.91
	Si12	1.624	4.03
	Si13	1.603	4.27
	Si14	1.612	4.15
	Si15	1.633	3.98
	Si16	1.599	4.33
$Na_6(UO_2)_3(Si_2O_7)_2$	Si1	1.602	4.24
$Na_2UO_2SiO_4$	Si1	1.603	4.22
$Ba(UO_2)(Si_2O_6)$	Si1	1.596	4.31
$Na_2(UO_2)_2(SiO_4)F_2$	Si1	1.607	4.18
$Na_2(UO_2)(Si_4O_{10})(H_2O)_{2.1}$	Si1	1.613	4.12
	Si2	1.611	4.12
$Cs_2(UO_2)Si_2O_6$	Si1	1.616	4.08
	Si2	1.627	3.97
$K(UO)Si_2O_6$	Si1	1.619	4.05
	Si2	1.619	4.05
	Si3	1.623	4.01
	Si4	1.619	4.05
$Rb_2(UO_2)(Si_2O_6)(H_2O)$	Si1	1.633	3.91
	Si2	1.631	3.93
	Si3	1.642	3.82
	Si4	1.636	3.88
$Cs_2(UO_2)(Si_2O_6)(H_2O)_{0.5}$	Si1	1.626	3.98
	Si2	1.629	3.95
	Si3	1.625	3.99
	Si4	1.621	4.03
$Rb_4(UO_2)_2(Si_8O_{20})$	Si1	1.619	4.06

	Si2	1.622	4.02
	Si3	1.629	3.95
	Si4	1.605	4.20
$Rb_2(UO_2)Si_2O_6$	Si1	1.618	4.07
	Si2	1.613	4.12
	Si3	1.613	4.12
	Si4	1.617	4.08
$Cs_2(UO_2)Si_2O_6$	Si1	1.621	4.03
	Si2	1.606	4.19
$(K_3Cs_4F)((UO_2)_3(Si_2O_7)_2)$	Si1	1.620	4.06
	Si2	1.634	3.90
$(NaRb_6F)((UO_2)_3(Si_2O_7)_2)$	Si1	1.628	3.97
$K_2Ca_4((UO_2)(Si_2O_7)_2)$	Si1	1.619	4.05
	Si2	1.616	4.08
$Cs_2UO_2Si_{10}O_{22}$	Si1	1.596	4.30
	Si2	1.614	4.10
	Si3	1.597	4.29
	Si4	1.598	4.28
	Si5	1.603	4.23
	Si6	1.604	4.21
	Si7	1.604	4.21
	Si8	1.611	4.15
	Si9	1.598	4.28
	Si10	1.598	4.28
$Cs_2UO_2SiO_4$	Si1	1.615	4.10
$K_4CaU(Si_2O_7)_2$	Si1	1.595	4.32
$K(UO)Si_2O_6$	Si1	1.611	4.14
$K_8(K_5F)U_6Si_8O_{40}$	Si1	1.612	4.13
	Si2	1.618	4.06
	Si3	1.616	4.09
	Si4	1.612	4.13
	Si5	1.634	3.90
	Si6	1.629	3.95
	Si7	1.620	4.04
	Si8	1.633	3.92

Table S12. Bond valences parameters in structure of $(K,Rb)_2[(UO_2)(Si_{10}O_{22})]$

	**O1**	**O2**	**O3**	**O4**	**O5**	**O6**	**O7**	**O8**	**O9**	**O10**	**O11**	**O12**	O13	O14	O15	O16	∑
**U1**	1.69×2					0.62×4											**5.84**
**U2**		1.70×2			0.62×4												**5.90**
**Si1**				1.03			1.04				1.10		1.04				**4.20**
**Si2**						1.11	1.00			0.99				1.05			**4.16**
**Si3**												1.06	1.13	1.13		1.11	**4.43**
**Si4**			1.09						1.05						1.14	1.14	**4.42**
**Si5**					1.10			0.99	1.01		1.04						**4.14**
**Rb1**[1]	0.10					0.09×2		0.12<br>0.04	0.05×2			0.04					**0.68**
**K1**		0.11			0.15×2	0.06×2	0.1×2			0.14<br>+0.05							**0.93**
∑	**1.79**	**1.81**	**2.18**	**2.06**	**1.87**	**1.88**	**2.14**	**2.1**	**2.11**	**2.12**	**2.14**	**2.16**	**2.17**	**2.18**	**2.28**	**2.25**	

Note. [1] The calculation was carried out using parameters for Rb-O bond. Rb1/K1 = 0.366(7)/0.634(7).

Table S13. Bond valences parameters in structure of $[Rb_3Cl][(UO_2)(Si_4O_{10})]$

	**O1**	**O2**	**O3**	**O4**	**O5**	**Cl1**	
**U1**	0.65×4		1.68×2				**5.94**
**Si1**	1.11	1.04		1.04	1.01		**4.20**
**Rb1**	0.11×4	0.08×2	0.06×2			0.2×2	**1.12**
**Rb2**	0.06×2		0.16		0.07×2	0.12×2	**0.66**
	**1.93**	**2.16**	**1.9**	**2.08**	**2.09**	**0.64**	

Table S14. Bond valences parameters in structure of $[Cs_3Cl][(UO_2)(Si_4O_{10})]$

	**O1**	**O2**	**O3**	**O4**	**O5**	**F1**	**F2**	**F3**	
**U1**		1.63×2	0.66×4						**5.90**
**Si1**	1.02		1.11	1.02	1.03				**4.18**
**Rb1**		0.08×2				0.12×2	0.1×2	0.07×2<br>0.08×2	**0.89**
**Rb2**		0.18	0.08×2		0.05×2	0.05×2			**0.57**
	**2.04**	**1.89**	**1.77**	**2.04**	**2.11**	**0.34**	**0.2**	**0.3**	
